# Transcriptomic Profile of Penicillium digitatum Reveals Novel Aspects of the Mode of Action of the Antifungal Protein AfpB

**DOI:** 10.1128/spectrum.04846-22

**Published:** 2023-04-06

**Authors:** Carolina Ropero-Pérez, Begoña Bolós, Moisés Giner-Llorca, Antonella Locascio, Sandra Garrigues, Mónica Gandía, Paloma Manzanares, Jose F. Marcos

**Affiliations:** a Department of Food Biotechnology, Instituto de Agroquímica y Tecnología de Alimentos (IATA), Consejo Superior de Investigaciones Científicas (CSIC), Paterna, Valencia, Spain; Centro de Investigaciones Biologicas CSIC

**Keywords:** antifungal proteins (AFPs), *Penicillium digitatum*, AfpB, transcriptomics, killing mechanism

## Abstract

Antifungal proteins (AFPs) from filamentous fungi are promising biomolecules to control fungal pathogens. Understanding their biological role and mode of action is essential for their future application. AfpB from the citrus fruit pathogen Penicillium digitatum is highly active against fungal phytopathogens, including its native fungus. Our previous data showed that AfpB acts through a multitargeted three-stage process: interaction with the outer mannosylated cell wall, energy-dependent cell internalization, and intracellular actions that result in cell death. Here, we extend these findings by characterizing the functional role of AfpB and its interaction with P. digitatum through transcriptomic studies. For this, we compared the transcriptomic response of AfpB-treated P. digitatum wild type, a Δ*afpB* mutant, and an AfpB-overproducing strain. Transcriptomic data suggest a multifaceted role for AfpB. Data from the Δ*afpB* mutant suggested that the *afpB* gene contributes to the overall homeostasis of the cell. Additionally, these data showed that AfpB represses toxin-encoding genes, and they suggest a link to apoptotic processes. Gene expression and knockout mutants confirmed that genes coding for acetolactate synthase (ALS) and acetolactate decarboxylase (ALD), which belong to the acetoin biosynthetic pathway, contribute to the inhibitory activity of AfpB. Moreover, a gene encoding a previously uncharacterized extracellular tandem repeat peptide (TRP) protein showed high induction in the presence of AfpB, whereas its TRP monomer enhanced AfpB activity. Overall, our study offers a rich source of information to further advance in the characterization of the multifaceted mode of action of AFPs.

**IMPORTANCE** Fungal infections threaten human health worldwide and have a negative impact on food security, damaging crop production and causing animal diseases. At present, only a few classes of fungicides are available due to the complexity of targeting fungi without affecting plant, animal, or human hosts. Moreover, the intensive use of fungicides in agriculture has led to the development of resistance. Therefore, there is an urgent need to develop antifungal biomolecules with new modes of action to fight human-, animal-, and plant-pathogenic fungi. Fungal antifungal proteins (AFPs) offer great potential as new biofungicides to control deleterious fungi. However, current knowledge about their killing mechanism is still limited, which hampers their potential applicability. AfpB from P. digitatum is a promising molecule with potent and specific fungicidal activity. This study further characterizes its mode of action, opening avenues for the development of new antifungals.

## INTRODUCTION

Filamentous fungi provide great biotechnological opportunities due to their diversity, exceptional metabolic capacity, and rapid ability to adapt to diverse ecosystems. However, fungi may also pose a challenge due to their capacity to infect a vast range of organisms and negatively affect food safety (1). Consequently, there is an urgent need to find antifungal molecules with novel modes of action and high specificity.

Fungal antifungal proteins (AFPs) have attracted much attention due to their potential to control pathogenic fungi (2, 3). AFPs are small, cysteine rich, and highly stable cationic proteins that are secreted by certain strains of ascomycete fungi, mainly from the genera *Penicillium* and Aspergillus. AFPs and AFP-derived peptides have already been demonstrated to effectively protect from fungal infections *in vivo* against economically relevant phytopathogens (4–8). However, current knowledge about the killing mechanism of AFPs is limited, which hampers their applicability. In this sense, a detailed understanding of the mode of action of AFPs is essential.

Penicillium digitatum is the main postharvest pathogen of citrus fruits (9), and the genomes of several strains have been sequenced (10–14), which sets the basis for in-depth molecular characterization. P. digitatum encodes only one AFP, which was named AfpB. The protein could not be detected in the wild-type strain of P. digitatum, although its encoding gene was transcribed at high levels (15). Later, homologous AfpB production was achieved (up to 20 mg/L) under the control of the regulatory elements of the P. chrysogenum *paf* gene that encodes the antifungal protein PAF (16–18). AfpB is a highly active protein, and its antifungal activity has been demonstrated against opportunistic human-, animal-, plant-, and foodborne pathogenic fungi, including its native fungus (4, 16, 19). Our previous cell biology studies showed that AfpB is a cell-penetrating protein that induces regulated cell death (20) through (i) interaction with the fungal cell wall, (ii) energy-dependent cell internalization, and (iii) cell collapse. Moreover, we determined that cell wall integrity-related genes are involved in P. digitatum sensitivity to AfpB and that this protein induces MAPK pathways related to cell wall and osmotic stress (20–22).

Several proteomic studies previously revealed the effect of Penicillium chrysogenum PgAFP on the proteome of other food-related fungi such as Aspergillus flavus, Penicillium polonicum, and Penicillium expansum, pointing to an increase of oxidoreductases, heat shock proteins, or cell wall-degrading enzyme abundance and, in particular cases, alteration of mycotoxin production (23–25). Additionally, a meta-analysis of transcriptomic data in Aspergillus niger suggested novel functions for its own AnAFP in nutrient recycling during starvation, autophagy, and cell development (26).

In this study, we provide additional data on the mechanism of action and biological role of AfpB on its own fungus. We have characterized the transcriptomic changes that occur in the P. digitatum wild type upon exposure to AfpB under different experimental conditions, as well as in an *afpB* disruption mutant (Δ*afpB*) (15) and in a P. digitatum strain that overproduces large amounts of the protein (17). As far as we are concerned, this is the first study that shows the transcriptomic response of a phytopathogenic fungus after different treatments with its self-inhibitory protein, which offers a rich source of information to advance in the characterization of the biological role and mode of action of antifungal proteins for future applications.

## RESULTS

### Improvement of the functional annotation of the P. digitatum genome.

Initial analyses on P. digitatum CECT 20796 (PHI26, wild type) genome (14) deposited at Joint Genome Institute (JGI) (https://mycocosm.jgi.doe.gov/Pendi1/) and UniProt (https://www.uniprot.org/proteomes/UP000009882) indicated that the functional annotation was not optimal. Annotations at JGI and UniProt have 45.5 and 34.7% of genes with no gene ontology (GO) terms, respectively, while only 16.5 and 12.3% of the genes have annotations in the three GO categories. To improve genome annotation, we used the functional annotation package within the OmicsBox suite, and we chose 11 extensively annotated genomes that are related to P. digitatum (see Table S1 in the supplemental material). We obtained improved functional annotation (see Materials and Methods), with only 7.8% of the genes lacking GO terms and more than 50% having now the three GO categories (Fig. S1). This improved annotation enhanced the analysis of functional enrichment of the genes discussed in this work, resulting in significant GO terms among the differentially expressed genes (DEGs) identified. The improved annotation is available through this study (Data Set S1).

### Transcriptomic profiling of the P. digitatum Δ*afpB* mutant.

**FIG 1:**
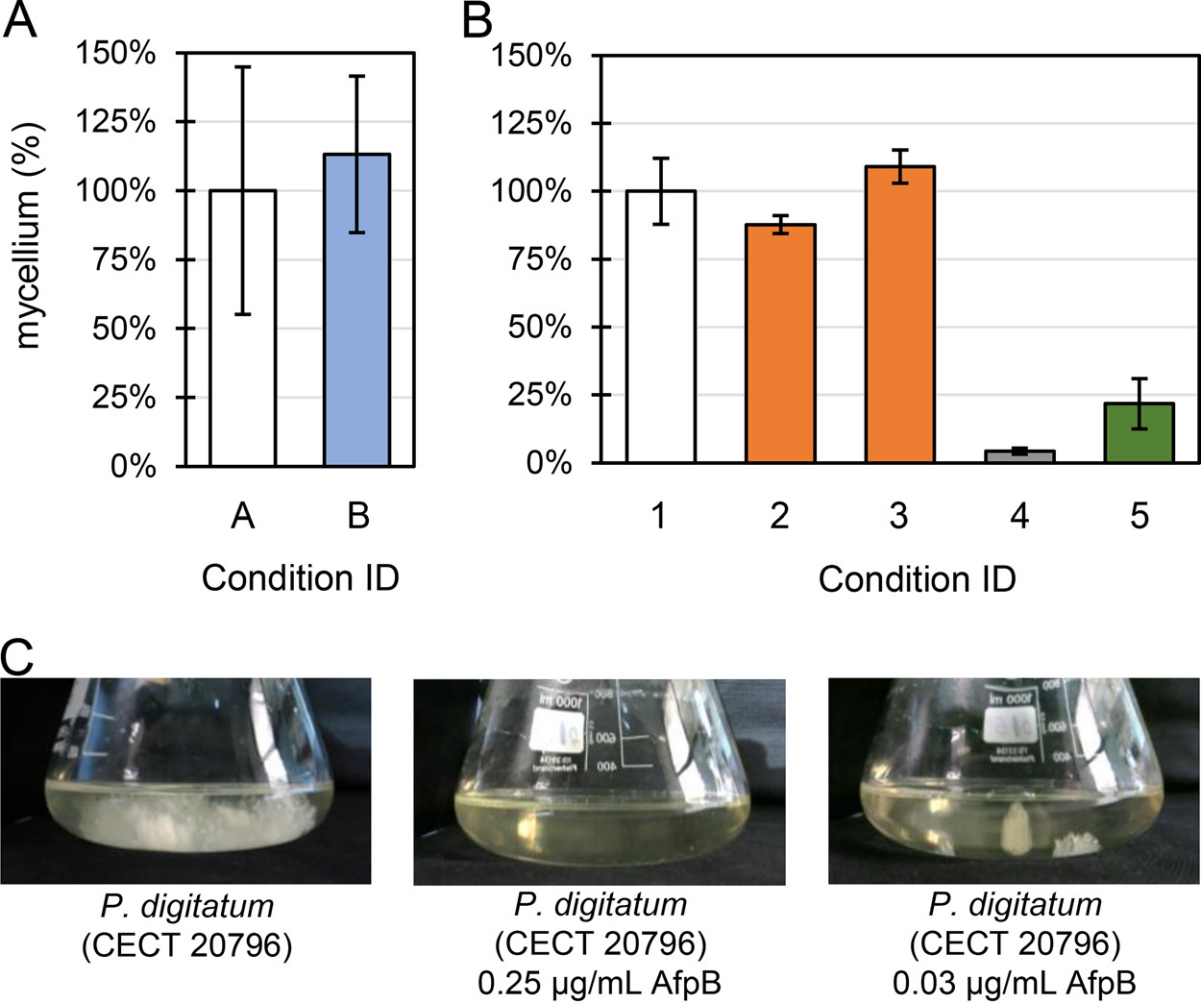
(A) Biomass recovery at experiment I after cultivation of P. digitatum CECT 20796 (condition A, *x* axis) and the Δ*afpB* strain PDMG122 (condition B). (B) Biomass recovery at experiment II under condition IDs 1 to 5 (Table 1) (*x* axis). ID 1, control, CECT 20796 in the absence of AfpB; IDs 2 to 3, CECT 20796 grown for 48 h in the absence of AfpB and then treated with 1 μg/mL of AfpB for 1 h (ID 2) and 3 h (ID 3); ID 4, CECT 20796 grown in the continuous presence of 0.03 μg/mL of AfpB; ID 5, AfpB-overproducing strain PDSG420. In panels A and B, data are the mean percentage of mycelium fresh weight recovery in the P. digitatum CECT 20796 (control conditions A and 1, respectively) ± standard deviation. (C) Images of flasks comparing the growth of P. digitatum CECT 20796 without AfpB or in the presence of 0.25 μg/mL or 0.03 μg/mL of AfpB.

To shed light on the mode of action and the biological role of AfpB, we first determined (Table 1, experiment I) the transcriptomic signature of the Δ*afpB* mutant PDMG122 (condition B) (15) compared to that of the wild-type CECT 20796 (condition A). We previously reported that PDMG122 had no phenotypic differences from its parental CECT 20796 (15), and, accordingly, we observed no growth differences under the conditions used (Fig. 1). Across four biological replicates, 0.80 ± 0.36 g of fresh mycelia were collected in the parental strain after 48 h of incubation (referred to as the relative value, 100% ± 45%), whereas 0.90 ± 0.23 g (113% ± 28%) were collected for the mutant (Fig. 1A).

**TABLE 1:** Conditions used in this study

ID	Strain	Growth (48 h, 25°C)	Treatment
Expt I			
A	P. digitatum CECT 20796	25% PDB	Control
B	P. digitatum PDMG122 (Δ*afpB*)	25% PDB	
Expt II			
1	P. digitatum CECT 20796	25% PDB	Control
2	P. digitatum CECT 20796	25% PDB	1 h, 1 μg/mL AfpB
3	P. digitatum CECT 20796	25% PDB	3 h, 1 μg/mL AfpB
4	P. digitatum CECT 20796	25% PDB + 0.03 μg/mL AfpB	
5	P. digitatum PDSG420 (AfpB^op^)	25% PDB	

**FIG 2:**
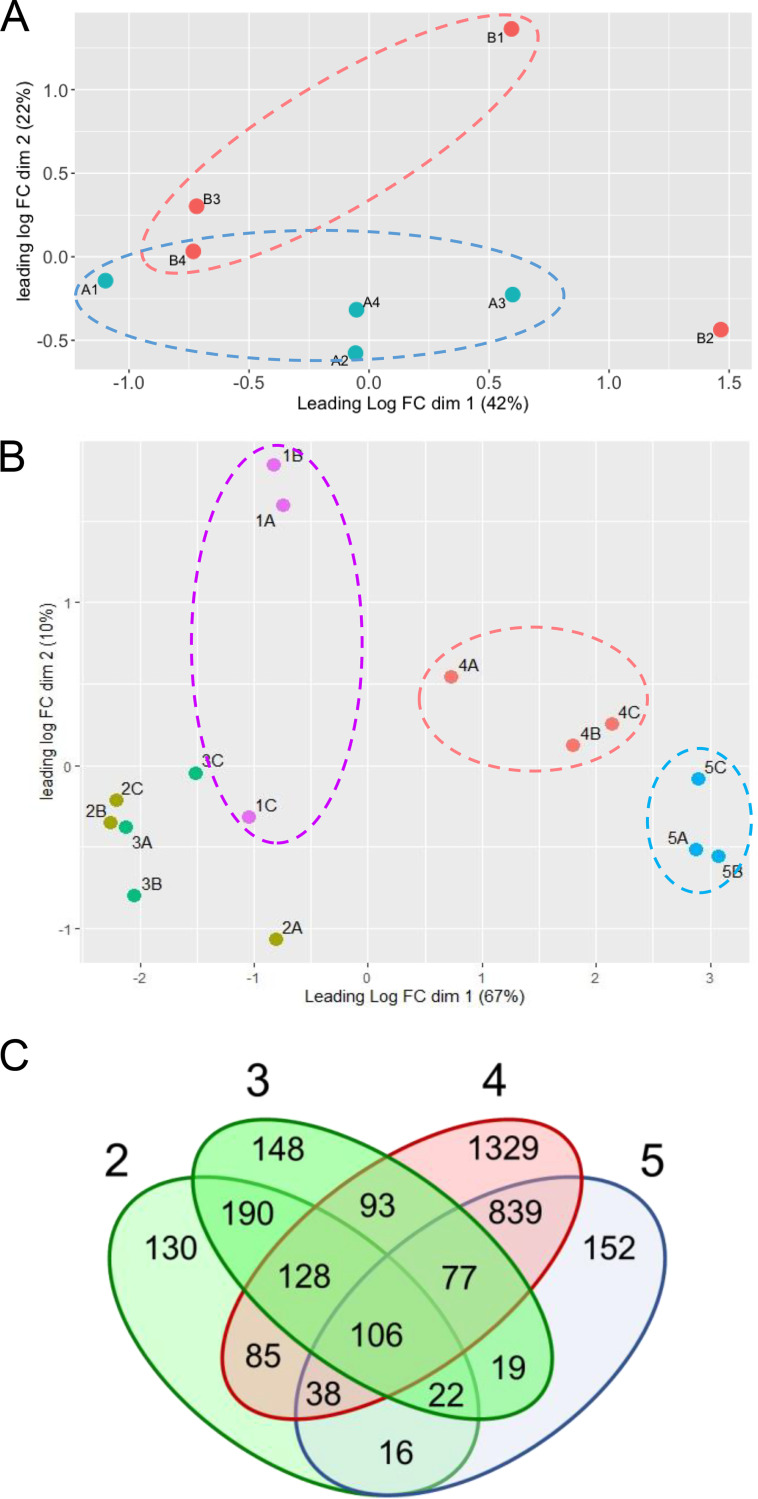
(A) Multidimensional scaling (MDS) plot of the RNA-seq expression profiles in experiment I. The conditions A and B (Table 1, experiment I) with four replicates under each condition (1 to 4) are plotted. (B) MDS plot of the RNA-seq expression profiles in experiment II. Conditions 1 to 5 (Table 1, experiment II) with three replicates under each condition (A to C) are plotted. The distances in the MDS plots represent the root mean square deviation (Euclidean distance) for the genes with the largest standard deviations between samples. (C) Venn diagram depicting shared and unique DEGs between each condition (2–5) compared to the control condition (1) in experiment II. Condition IDs are as follows: B, P. digitatum PDMG122; 2, CECT 20796 grown for 48 h and then treated with 1 μg/mL AfpB for 1 h; 3, CECT 20796 grown for 48 h and then treated with 1 μg/mL AfpB for 3 h; 4, CECT 20796 grown in the continuous presence of 0.03 μg/mL AfpB; and 5, P. digitatum PDSG420.

Transcriptome sequencing (RNA-seq) analysis across four biological replicates of each strain revealed a total number of uniquely mapped reads, between 22.2 and 31.1 million reads per sample. The number of reads that mapped to annotated transcripts ranged between 16.6 and 23.8 million reads, equivalent to a relative amount between 74.60 and 76.58% (Table S2). Normalized count data were subjected to principal-component analysis (PCA) (Fig. 2A). Despite the discrepancy showed by one replicate, we decided to keep it in the analysis given the biological variability associated with filamentous fungal growth and considering the application of more restrictive conditions for the identification of specific DEGs associated with *afpB* disruption.

A total of 8,148 genes (89.2% of the 9,132 genes of the P. digitatum CECT 20796 genome) were above the threshold required for differential expression analysis, and 211 (2.3%) were considered DEGs in the Δ*afpB* mutant. A total of 177 of these DEGs were downregulated, while 34 were upregulated (Data Set S2).

Functional enrichment analysis of GO terms among the DEGs of experiment I is shown in Fig. S2. Among the downregulated DEGs in the Δ*afpB* mutant, the terms with the highest enrichment factor (overrepresented) in the three GO categories are related to maturation of rRNA and assembly of ribosomes and genes related to RNA polymerase I, II, and III, indicating a general repression of transcription and ribosome biogenesis. The only GO term significantly overrepresented in the upregulated DEGs was the phosphopantetheine binding (GO:0031177), consisting of four induced genes (PDIG_09960, PDIG_55700, PDIG_41980, and PDIG_39590) with annotations related to nonribosomal peptide (NRP) synthetases and toxins. For instance, the gene PDIG_09960 is homologous to *tqaA* from Penicillium aethiopicum, which is involved in the biosynthesis of toxic indol alkaloids (27). P. digitatum genes mentioned throughout the text are specifically tagged (shown as “#”) in Data Set S2.

### Large culture volumes with strong aeration result in higher apparent antifungal activity of AfpB against P. digitatum.

Another objective of this study was to determine the transcriptomic response of P. digitatum wild type to AfpB (Table 1, experiment II). For this aim, two approaches were designed, (i) pregrown mycelium treated with subinhibitory concentrations of AfpB (1 μg/mL) for 1 or 3 h (conditions 2 to 3), or (ii) fungal growth in the continuous presence of AfpB (condition 4). Our previous data indicated that the MIC of AfpB against P. digitatum in microtiter plates is 4 μg/mL (16), which conceptually matches condition 4. However, under the growth conditions of this study (200 mL culture volumes under strong aeration), we found that AfpB exerts much higher inhibitory activity. Concentrations as low as 0.25 μg/mL (40 nM) and 0.065 μg/mL (10 nM) completely inhibited measurable growth (Fig. 1C and data not shown), which is more than 50 times below the MIC in microtiter plates. In the presence of 0.03 μg/mL (5 nM) AfpB (condition 4), only 4% of the biomass was recovered compared with the untreated control (0.05 ± 0.01 g versus 1.09 ± 0.13 g) (Fig. 1B and C). On the other hand, when fungal cultures were pregrown for 48 h and then treated with 1 μg/mL AfpB for 1 or 3 h, we observed no macroscopic change (Fig. 1B).

In parallel, we determined the transcriptome of P. digitatum AfpB-overproducing strain PDSG420 (condition 5). This strain, described previously, registered a slightly slower growth in solid medium than the wild type (17). However, the culture conditions of this study resulted in a growth penalty for this strain since the biomass recovered barely reached 25% (Fig. 1B). Taking all results together, we conclude that large-volume cultures under strong aeration result in distinct antifungal potency of AfpB and differences in the effect of the *afpB* gene overexpression on P. digitatum.

### Transcriptomic response of P. digitatum to AfpB.

All conditions from experiment II (Table 1) were subjected to transcriptome analyses. The lowest and highest numbers of reads across the samples were 21.7 and 40.6 million, respectively. The percentage of reads that mapped to the genome was between 91.8 and 94.0%. The total number of reads that mapped to annotated transcripts ranged between 14.9 and 26.1 million reads, equivalent to a relative percentage between 65.32 and 79.47% (Table S2).

Normalized data were subjected to PCA. As shown in Fig. 2B, dimension 1 explains 67% of the sample variability, whereas dimension 2 explains 10%. Results indicate that conditions 1, 2, and 3 are closely grouped within dimension 1, differentiating from conditions 4 and 5 (Fig. 2B). Additionally, two of the biological replicates from the control (condition 1) were clearly separated from the other conditions in dimension 2, while the third one grouped with conditions 2 and 3. These results may indicate that the transcriptomic response of the parental strain in the continuous presence of AfpB is close to the transcriptomic response of the AfpB-overproducing strain PDSG420 but clearly distinguishes it from the response after short-term AfpB treatment or no treatment. Despite the discrepancy shown by one of the replicates in the control, we decided to keep it in the analysis, assuming that it would result in more restrictive conditions for the identification of specific responses.

A total of 91.8% of the genes annotated in P. digitatum (8,387 genes out of the 9,132) passed the filtering for DEG analysis. Figure 2C shows the Venn diagram depicting the shared and unique DEGs under each condition of experiment II. Condition 4 showed the highest number of DEGs (2,695, 32.1%), including the highest number of DEGs exclusive for this condition (1,329, 15.8%), followed by condition 5 (152, 1.8%). As expected from the PCA, the growth of the parental strain in the presence of AfpB and the AfpB overproducer (conditions 4 and 5) share 839 DEGs that were not differentially expressed in the rest of the conditions. In contrast, the numbers of DEGs in conditions 2 and 3 were lower than in 4 and 5. In fact, only 130 and 148 DEGs, respectively, were exclusive for each of the two conditions, sharing 190 DEGs that were not represented in conditions 4 and 5.

Out of the 8,387 genes analyzed, only 106 (1.2%) are common DEGs in the four conditions tested (Fig. 2C). These genes may constitute a “core” that defines the effect of the protein on the fungus. Unfortunately, GO analyses did not show any significant annotation in this gene set at false-discovery rate (FDR) of <0.05. Interestingly, among this group, PDIG_14840, coding for acetolactate synthase (ALS), and PDIG_14850, coding for acetolactate decarboxylase (ALD), are located in a cluster of five genes (PDIG_14820 to PDIG_14860) that are highly responsive to the protein. PDIG_14840 and PDIG_14850 showed induction after short times of AfpB exposure (conditions 2 to 3) but strong repression in the continuous presence of the protein (condition 4) and the overproducer (condition 5). Additionally, 7 of these 106 DEGs (6.6%) are annotated as major facilitator superfamily (MFS) domain transporters or amino acid/peptide transporters. Five of them were repressed under all the conditions of experiment II. However, the other two (the MFS-encoding PDIG_38440 and PDIG_64980) showed high induction after short times of AfpB exposure (conditions 2 and 3) but were repressed under conditions 4 and 5. Additional peptide transporter genes responded to some of the treatments in this experiment, such as PDIG_34470 and PDIG_70130, which are among the most induced genes of the genome after 3 h treatment with AfpB.

### Comparison of the transcriptomic signatures across both experiments and all conditions.

**FIG 3:**
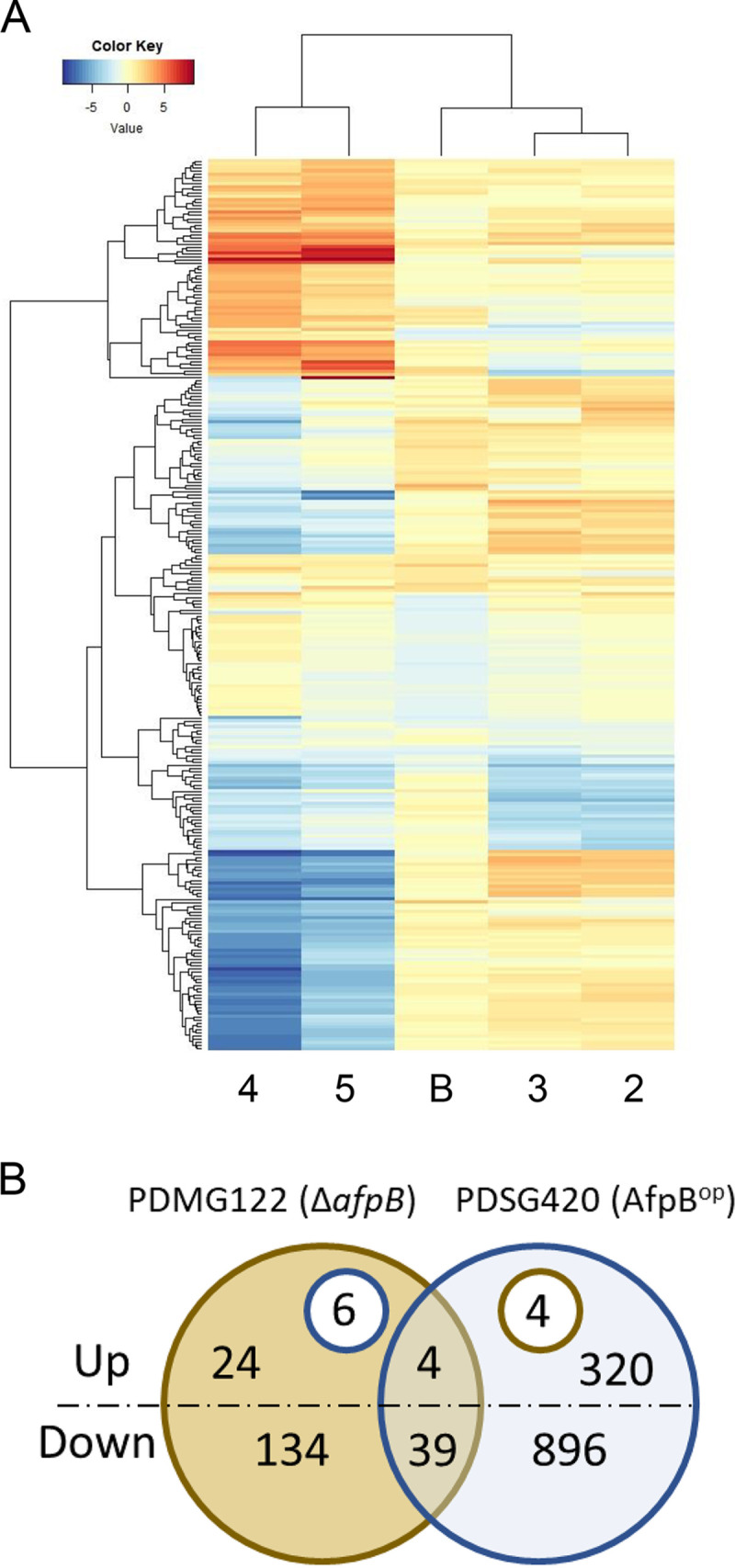
(A) Heatmap showing top differentially expressed genes (DEGs) for experiments I and II sorted by log fold change (FC). The 100 most variable DEGs for each individual condition (experiment II, conditions 2, 3, 4, and 5; experiment I, condition B) compared to the control in each experiment were selected to represent the heatmap. Hierarchical clustering is included for the conditions (*x* axis) and genes (*y* axis) with similar expression profiles. Condition ID 2, CECT 20796 grown for 48 h and then treated for 1 h with 1 μg/mL AfpB; 3, CECT 20796 grown for 48 h and then treated for 3 h with 1 μg/mL AfpB; 4, CECT 20796 grown in the continuous presence of 0.03 μg/mL AfpB; 5, P. digitatum PDSG420; B, P. digitatum null *afpB* PDMG122. (B) Venn diagram comparing common and unique upregulated (up) and downregulated (down) genes in the *afpB*-null strain (PDMG122) and the AfpB-overproducing strain (PDSG420). The insets within the upregulated DEGs indicate those that are upregulated in that strain but downregulated in the other.

Data Set S2 shows the 9,132 genes in P. digitatum CECT 20796 genome and their expression pattern across all conditions in the two experiments (I and II) and therefore provides an overview of the results of this study. In this line, Fig. 3A shows a heatmap of the 100 most DEGs for each condition tested (296 genes) with clusters’ “conditions” (horizontal axis) and “genes” (vertical axis) with similar expression profiles.

Overall, the expression fold changes in the Δ*afpB* mutant (condition B, experiment I) were not as high as those of experiment II. The most induced gene in our study was the AfpB-encoding gene (PDIG_68840, *afpB*; Data Set S2) in the AfpB overproducer (condition 5). In contrast, this gene was repressed when the fungus grew in the continuous presence of AfpB (condition 4). Neither induction nor repression of *afpB* was detected after short AfpB treatments (conditions 2 to 3). Following *afpB*, PDIG_81760 was the second most induced gene in our study, being the most induced in condition 4, and showing an induction of log fold change (logFC) above 8 under both 4 and 5 (Data Set S2). This gene annotated as “hypothetical protein” encodes a putative secreted and anionic peptide with a remarkable repetitive structure (see below). PDIG_81760 is located in a region of six contiguous genes that are the most induced by AfpB (PDIG_81740 to PDIG_81790).

On the other hand, among the most downregulated genes across our study, we found a few DEGs that were strongly repressed under continuous exposure to AfpB (condition 4) and in the overproducing strain (condition 5) but, remarkably, showed induction after short exposure to the protein (conditions 2 and 3). These include the terpenoid synthase-encoding PDIG_50820; PDIG_14840 and PDIG_14850, both involved in the acetolactate metabolism (see below); and the endopolygalacturonase-encoding PDIG_50670.

Only seven genes were DEGs under all five conditions across experiment I and II. These are the induced hydrolase-encoding PDIG_50070; and the repressed helicase-encoding PDIG_47930 and PDIG_29490, the reductase-encoding PDIG_83040, PDIG_49620, PDIG_33190, and the RNA processing-involved PDIG_79790 (Data Set S2). However, since these seven genes had the same expression pattern under all conditions, including in the Δ*afpB* mutant PDMG122, no clear relationship of these genes can be attributed to the AfpB mechanism of action or biological role.

In Fig. 3B, we compared the number of DEGs in the two genetically modified strains used, the null mutant and the AfpB overproducer. The few DEGs that have an opposite behavior in these two strains may help in the identification of the mode of action of AfpB. Most of them are confirmed in the continuous presence of AfpB. Thus, out of the six genes that are upregulated in the null mutant and downregulated in the overproducer, five of them are also downregulated under condition 4 (PDIG_01240, PDIG_05300, PDIG_23670, PDIG_50070, and PDIG_64860), while the four genes that are downregulated in the null mutant and upregulated in the overproducer are confirmed under the same condition (PDIG_08230, PDIG_47410, PDIG_67250, and PDIG_87540) (Data Set S2). Unfortunately, there is no obvious link among the annotations of these genes, although future work will attempt to deepen their significance. In this context, it is noteworthy that the DEG with the highest induction in the null mutant was PDIG_07370, coding for an inhibitor of apoptosis-promoting Bax1 domain-containing protein. This gene was downregulated in the continuous presence of AfpB (condition 4), although did not show differential expression in the overproducer (condition 5).

### Penicillium digitatum mutants in the acetoin biosynthetic pathway are more tolerant to AfpB.

**FIG 4:**
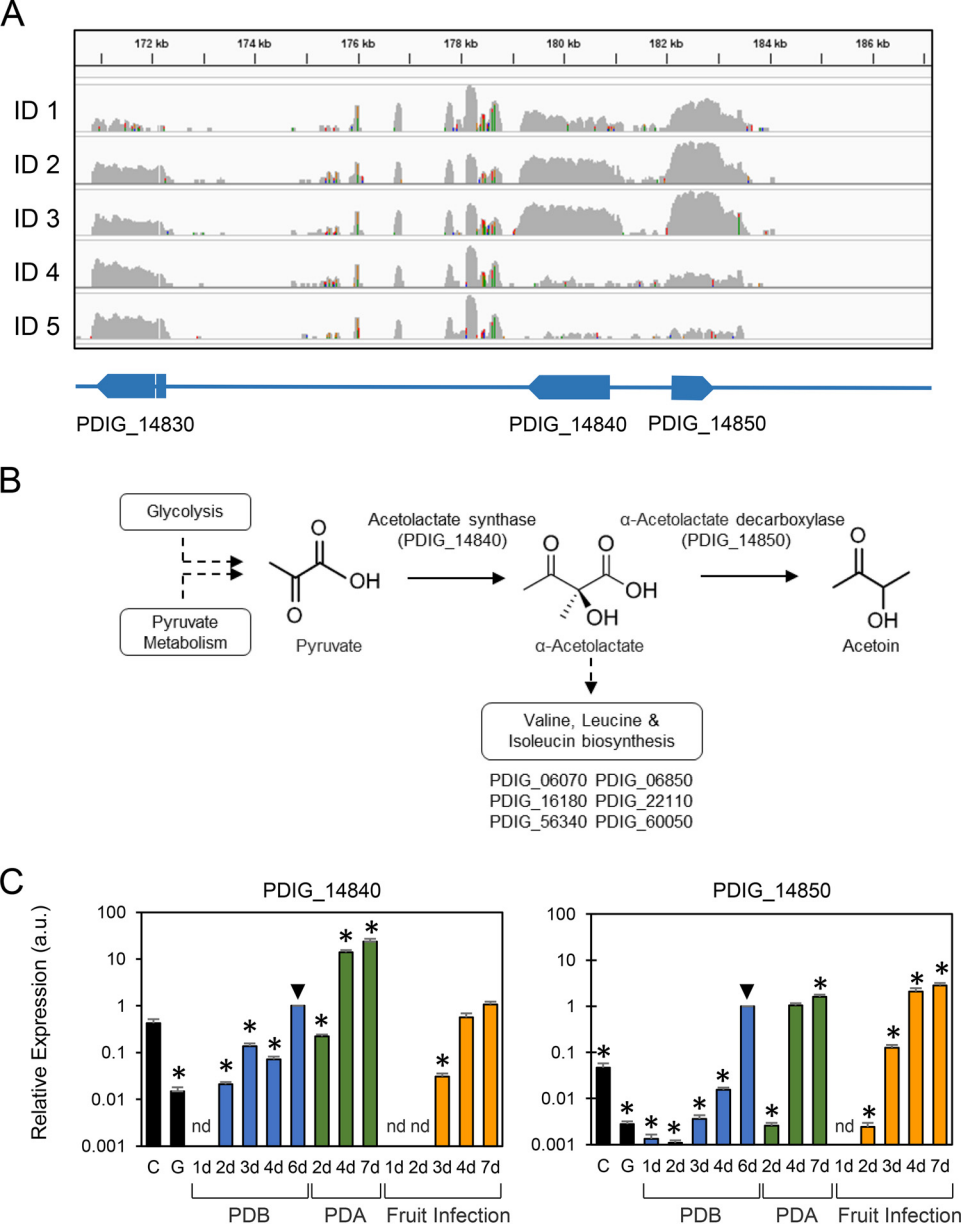
(A) Mapping of the RNA-seq reads in the genomic region of the acetoin biosynthetic cluster (PDIG_14840 and PDIG_14850) in condition IDs 1 (control) to 5 of experiment II (shown on the left; see also Table 1). (B) Schematic representation of the acetoin biosynthetic pathway and the ramification to the branched-chain amino acid biosynthetic pathway. Below the bifurcation, there is a list of the six P. digitatum genes that have been identified in this pathway. (C) Relative gene expression determined by qRT-PCR of PDIG_14840 and PDIG_14850 under different conditions of the biological cycle of the fungus; (*x* axis), quiescent (C) and germinating (G) conidia, axenic growth in liquid broth (PDB), solid medium (PDA), or during the infection process of citrus fruit at different days (d). The reference condition for the relative gene expression calculation was arbitrarily selected as growth in PDB for 6 days and is indicated with a black triangle. The relative quantification and statistical significance were determined by REST MCS v2 and REST 2009 software, respectively. Statistically significant differences from the 6-day reference (downward black triangle) are labeled with an asterisk (*P* < 0.05). a.u., arbitrary units.

As mentioned above, PDIG_14840 and PDIG_14850 were among the most AfpB-responsive genes in experiment II (Fig. 4A) (Data Set S2). PDIG_14840 encodes the large catalytic subunit of ALS, whose product is a common precursor in the biosynthesis of both acetoin and branched-chain amino acid pathways (Fig. 4B). PDIG_14850 encodes an ALD, which produces acetoin by acetolactate decarboxylation. Both PDIG_14840 and PDIG_14850 were induced upon short-term treatment with AfpB (conditions 2 to 3) but were strongly repressed under the continuous presence of the protein (condition 4) and in the overproducer (condition 5) (Fig. 4A). We further characterized the expression of these two genes during the biological cycle of the fungus by quantitative reverse transcription-PCR (qRT-PCR) (Fig. 4C), and in both cases, we detected the corresponding mRNAs in quiescent and germinating conidia. Moreover, their induction was maximum at late times of axenic growth and during fruit infection. However, their gene expression profiles were qualitatively different, with PDIG_14840 highly induced during growth on potato dextrose agar (PDA) plates, whereas PDIG_14850 showed a remarkable early expression at the onset of infection.

Additional downstream genes from the branched-chain amino acid biosynthetic pathway were identified in the P. digitatum genome (Fig. 4B), but they did not show noticeable expression changes. This would suggest that the part of the route that diverges to acetoin is the one involved in the mode of action of AfpB. To test this, null single PDIG_14840 and PDIG_14850 mutants, as well as the double mutant, were generated and verified (Fig. S3), and their growth, pathogenicity, and sensitivity to AfpB were characterized. Null mutants of PDIG_14840 (PDGL1711 and PDGL1712) and PDIG_14850 (PDGL0921 and PDGL0922), as well as the double mutants (PDGL1721 and PDGL1723), showed no growth difference in PDA (Fig. S4). Similarly, none of the mutants showed phenotypical differences during fruit infection (Fig. S4).

**FIG 5:**
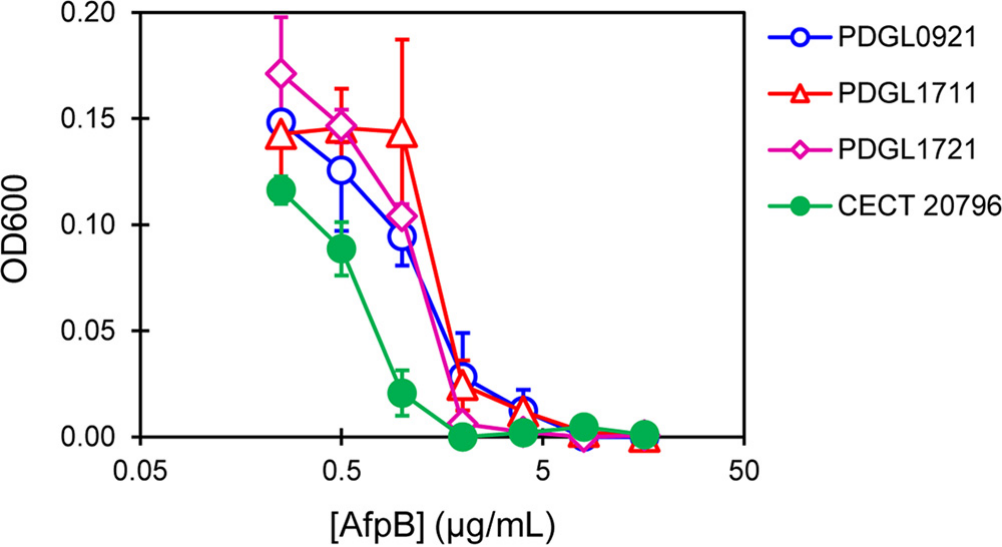
Dose-response curves of growth inhibition of P. digitatum wild-type strain CECT 20796 and the deletion mutant strains PDGL0921 (ΔPDIG_14850), PDGL1711 (ΔPDIG_14840), and PDGL1721 (ΔPDIG_14840 ΔPDIG_14850) by AfpB. Curves show the means ± SD of optical density at 600 nm (OD_600_) from triplicate samples at each AfpB concentration after 72 h of incubation.

Antifungal assays showed that single and double mutants in PDIG_14840 and PDIG_14850 presented subtle, but reproducible, increased tolerance (2-fold MIC increase) to AfpB, demonstrating a link between these genes and the mode of action of the protein (Fig. 5).

### The gene PDIG_81760 is highly responsive to AfpB and encodes a novel protein with an unusual structure.

**FIG 6:**
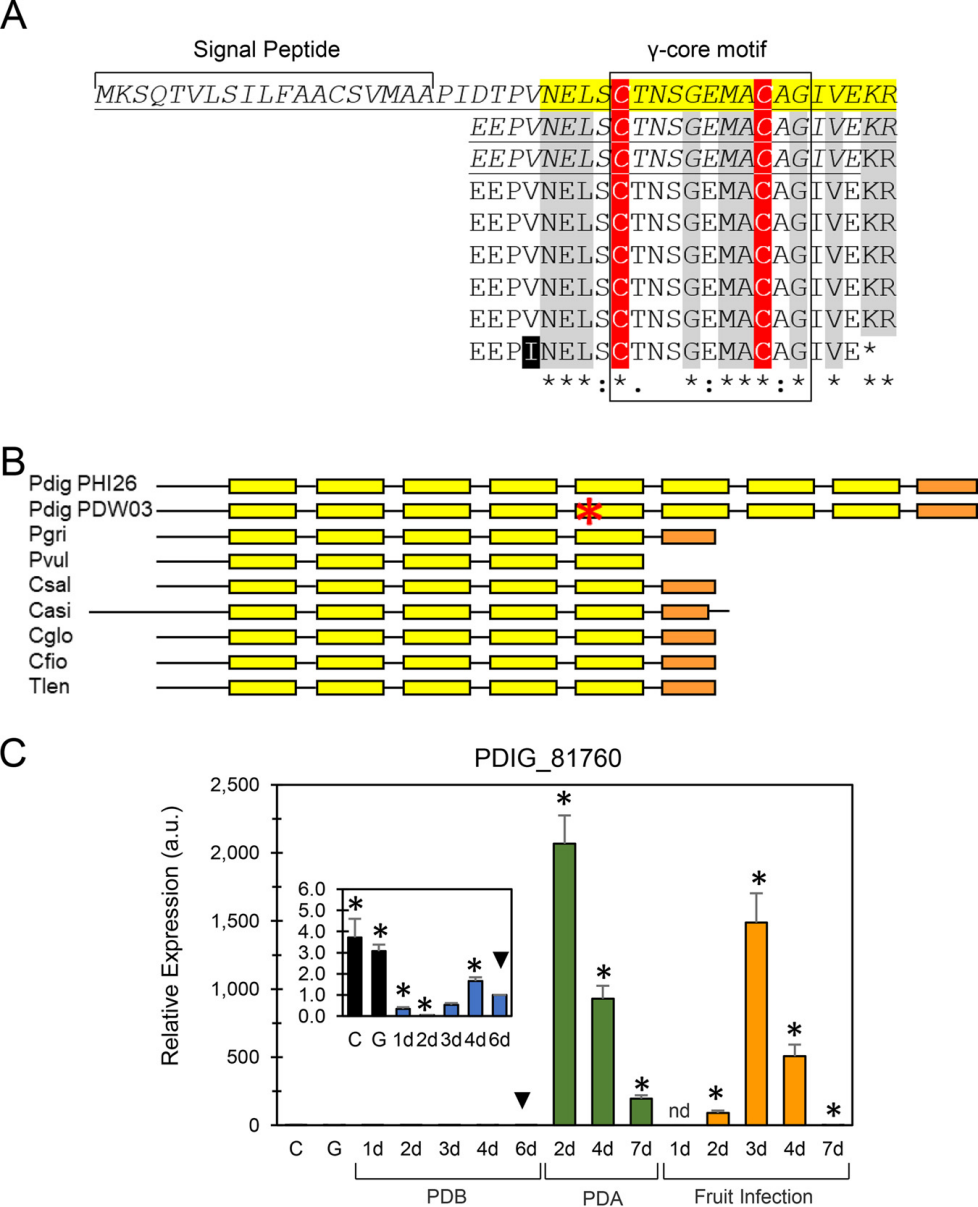
(A) Sequence of the putative TRP encoded by PDIG_81760. Repetitions are highlighted in yellow and aligned vertically. Conserved residues between other filamentous ascomycetes are highlighted in gray. Conserved cysteines are highlighted in red. (B) Schematic representation of the protein encoded by PDIG_81760 and its homologous proteins from different fungi (see the complete sequence alignment and ID of sequences in Fig. S5 in the supplemental material). Each repeat is colored in yellow in correlation with panel A. The last repeat is colored in orange to indicate the truncation of the last two residues. Note that two different P. digitatum sequences are depicted that correspond to the strain and sequence used in this work (Pdig_PHI26) and the completed genome sequence of strain PDW03. The red asterisk indicates the position of different amino acids in the conserved region of these two P. digitatum strains. (C) Expression pattern by qRT-PCR of PDIG_81760 under different conditions, quiescent (C) and germinating (G) conidia, axenic growth in liquid broth (PDB) and solid medium (PDA), and during the infection of citrus fruit. The relative quantification and statistical significance were determined by REST MCS v2 and REST 2009 software, respectively. Statistically significant differences from the 6-day reference (downward black triangle) are labeled with an asterisk (*P* < 0.05).

We next focused on the above-mentioned gene, PDIG_81760, which was highly induced when the fungus was exposed to high AfpB concentration and in the AfpB-overproducing strain (conditions 4 and 5, respectively) (Data Set S2). In contrast, PDIG_81760 was not significantly affected in the null mutant (condition B, experiment I). Remarkably, the initial mapping of the RNA-seq reads to PDIG_81760 showed single nucleotide polymorphisms that made us hypothesize some errors in the shotgun sequencing and/or assembly. We thus manually reanalyzed the genomic sequence through PCR amplification, cloning, and sequencing. Results showed that the locus was not correctly assembled and annotated. In fact, this gene corresponds to a longer repetition of a given peptide subunit. PDIG_81760 encodes a cysteine-rich anionic protein constituted by a tandem repeat peptide (TRP), preceded by a signal peptide for secretion (Fig. 6A). A BLAST protein similarity search showed that this gene is conserved in a specific group of filamentous ascomycetes (Fig. S5). PDIG_81760 homologs encode proteins that contain between five to nine repeats of a 20-amino-acid sequence (NEL[A/S]C[X_3_]G[E/D]MAC[A/S]GXVXKR) interlinked with 1 to 4 residues that contain, in most cases, one proline (Fig. 6A and B, yellow). The repeats are separated by the Kex2 protease recognition sequence (KR) (28). In most homologs, the last TRP does not contain the terminal KR. Instead, the last repeat is truncated by two residues (orange color in Fig. 6B). Each TRP includes a conserved levomeric isoform 1 of the γ-core motif (C[X_3-9_]CXG), which has been involved in structural stabilization of cysteine-rich proteins (CRPs) with antimicrobial activity (29).

PDIG_81760 had a remarkable expression pattern during the fungal biological cycle (Fig. 6C). We observed a low expression during growth in liquid medium, having the lowest expression level at the onset of growth (Fig. 6C). At early times during growth on solid surface (i.e., 2 days in PDA or 3 days during fruit infection), the gene expression peaked to 1,500- to 2,000-fold over the reference value. Later on, during aerial colonization, the expression declined. Following these observations, we generated null PDIG_81760 mutants (Fig. S6) and characterized them during axenic growth and fruit infection (Fig. S7). The two null PDIG_81760 mutants PDBB001 and PDBB005 showed no differential phenotypes during growth in PDA. A pathogenicity test suggested a slight decrease in virulence in citrus fruit. However, this was either not significant across all postinoculation days or was not confirmed in independent experiments (see Fig. S7C as a representative experiment).

The anionic protein encoded by PDIG_81760 could potentially antagonize the action of the cationic AfpB. We used the null mutants to test this hypothesis but found that the deletion of PDIG_81760 did not alter the sensitivity of the fungus to AfpB (Fig. S7D).

### Synthetic TRP monomers from the protein encoded by PDIG_81760 modulate AfpB activity *in vitro*.

**FIG 7:**
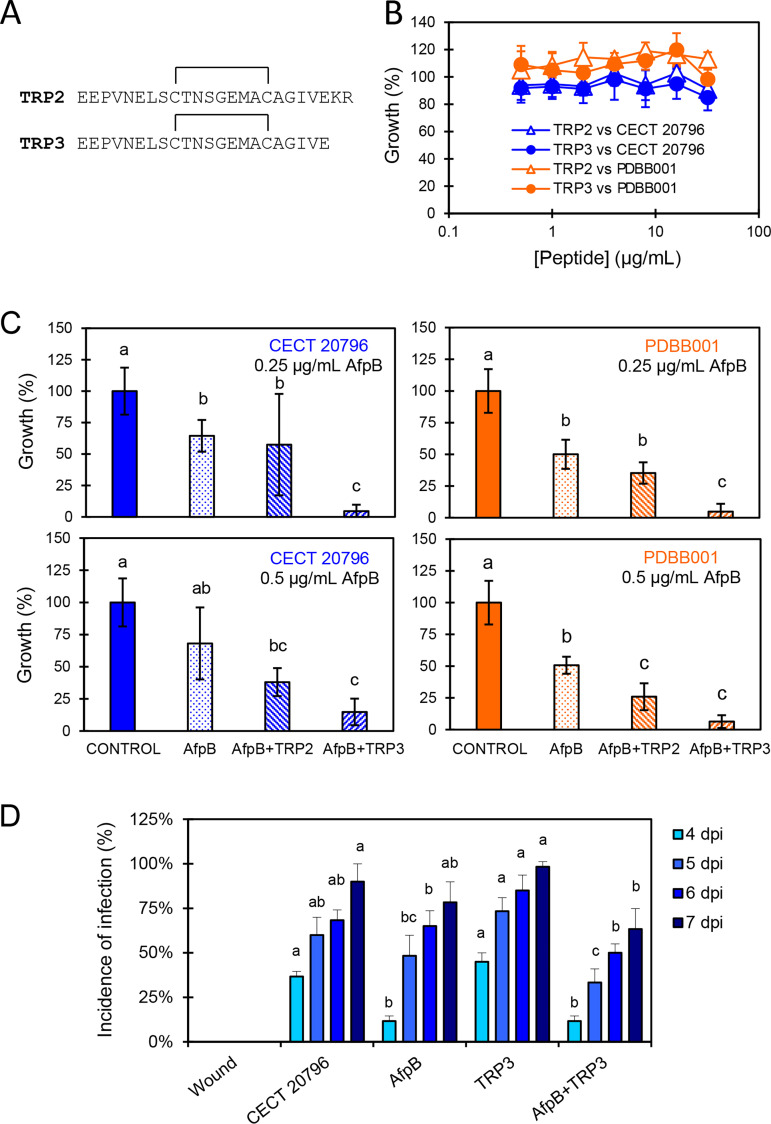
(A) Amino acid sequence of the synthetic peptides TRP2 and TRP3. Black lines show disulfide bridges between cysteine residues. (B) Dose-response curves showing the antifungal activity of TRP2 and TRP3 against P. digitatum CECT 20796 and the deletion mutant PDBB001. (C) Antifungal activity assay of AfpB (0.25 or 0.5 μg/mL) and its combination with TRP2 or TRP3 (16 μg/mL) against CECT 20796 and PDBB001. (D) Effect of AfpB, TRP3, and their combination on the infection of oranges by P. digitatum. Bars show the mean values of the percentage of infected wounds and standard deviation (SD) of three replicates of five oranges at 4, 5, 6, and 7 dpi. Letters show significant differences among the treatments on each independent day (one-way ANOVA and Tukey’s HSD test, *P *< 0.05).

Under the hypothesis that the constituent TRPs of PDIG_81760 are processed *in vivo*, we tested whether these monomeric peptides would affect either fungal growth or AfpB antifungal activity. We used the synthetic peptides TRP2 (which accounts for the perfect tandem repetition of PDIG_81760) and TRP3 (corresponding to the truncated terminal repeat in PDIG_81760), which are flanked by Kex2 recognition sites, and presumed that disulfide bonds are formed within the cysteines in each monomer (Fig. 7A). Neither TRP2 nor TRP3 showed any effect against the growth of the parental P. digitatum or the null PDBB001 mutant *in vitro* at the highest concentration tested (32 μg/mL) (Fig. 7B). However, when sub-MICs of AfpB were combined with 16 μg/mL of either TRP2 or TRP3, the antifungal activity of AfpB increased, showing almost complete inhibition at protein concentrations as low as 0.25 μg/mL in both wild-type and PDBB001 strains (Fig. 7C). In most experiments, TRP3 induced a higher AfpB activity than TRP2. Therefore, we aimed to determine if TRP3 would also improve AfpB activity *in vivo.* However, fruit inoculation experiments revealed that the addition of TRP3 to the AfpB treatment did not significantly improve the control of the *Penicillium* decay of citrus fruits (Fig. 7D).

## DISCUSSION

In this work, we performed a transcriptomic study to obtain information about the response of P. digitatum to its inhibitory protein AfpB and its role in the biological cycle of the fungus. When P. digitatum is treated with AfpB, the protein behaves as a cell-penetrating protein that triggers a regulated cell death (RCD) program through a three-step mechanism that includes interaction with the outer cell layer, internalization, and intracellular effects (20). These latter include the generation of reactive oxygen species (ROS), which induce expression of RCD-associated genes such as metacaspases and proapoptotic genes (20). The finding that AfpB elicits a program of self-elimination suggests that its biological role goes beyond defense against competitors. With the transcriptomic analysis presented here, we provide further information about the biological function of AfpB and its complex mode of action.

Several studies have shown the effect of antifungal agents on the transcriptome and/or proteome of sensitive fungi, providing clues to their mode of action. Transcriptomic studies of P. digitatum treated with essential oils revealed DEGs involved in RNA transcription and transport, ABC transporters, MAPK signaling, oxidative phosphorylation, biosynthesis of secondary metabolites, sulfur, nitrogen, fatty acid metabolism, and steroid biosynthesis (30). Similarly, Botrytis cinerea exposed to these compounds resulted in the differential expression of genes related to biosynthesis of secondary metabolites, amino acid, carbohydrate, and lipid metabolisms, as well as genes involved in transcription, replication, and genetic repair (31).

On the other hand, omics studies predicted functions for A. niger AnAFP (26) or for P. chrysogenum PgAFP (25) other than those purely related to their antimicrobial nature. A meta-transcriptomic analysis indicated the importance of AnAFP for the survival of its producing fungus during nutrient starvation, its interaction with the autophagic machinery, and the relationship of *anafp* with genes related to osmotic stress, developmental processes, and secondary metabolism (26). Proteomic studies from P. expansum treated with PgAFP showed changes in the content of proteins related to mycotoxin biosynthesis and osmotic stress due to the ability of PgAFP to induce ROS and pathogenicity- and virulence-related proteins (25).

In this and previous work, we showed that P. digitatum Δ*afpB* mutants do not have any differences regarding axenic growth or pathogenesis (Fig. 1) (15). Yet transcriptomic data indicated that *afpB* is required for the homeostasis of the cell since its disruption resulted in repression of the ribosome biogenesis, transcription, and transport of organic acids. Our results also showed that one of the most highly induced genes in the Δ*afpB* strain is PDIG_07370, whose improved annotation indicates homology with inhibitors of apoptosis-promoting Bax1 domain-containing proteins. The BAX family members mediate the permeabilization of the outer mitochondrial membrane and the subsequent release of apoptogenic molecules that lead to caspase activation (32). PDIG_07370, in contrast, was downregulated in the continuous presence of AfpB. These results indicate that AfpB represses an inhibitor of apoptosis and suggest a relationship between AfpB and the apoptotic processes similar to that described for AnAFP.

Additionally, four DEGs involved in the biosynthesis of NRP and/or HC toxin-like synthetases were overexpressed in the Δ*afpB* mutant and belong to the only significant GO term among the induced genes under this condition (GO:0031177). Three of them (PDIG_09960, PDIG_41980, and PDIG_55700) were conversely repressed in the fungus cultured in the presence of AfpB. Taken together, these data suggest that these genes are specifically repressed by AfpB. PDIG_41980 and PDIG_55700 are located next to membrane transporters (PDIG_41990 and PDIG_55710), which could transport toxins and were also repressed by AfpB. PDIG_09960 is homologous to the Penicillium aethiopicum *tqaA* gene in the cluster of 13 genes involved in the tryptoquialanine biosynthetic pathway (27). The 16 genes that surround PDIG_09960 (from PDIG_09840 to PDIG_09990) were coordinately repressed in the presence of AfpB (see Data Set S2 in the supplemental material), being the largest set of continuous genes that showed the same expression pattern in all of our studies. Additionally, 8 of them were repressed in the AfpB-overproducing strain. Tryptoquialanines are phytotoxic indole alkaloids, which are produced by P. digitatum in culture and during infection (33, 34). The involvement of tryptoquialanine A in citrus fruit infection was evaluated through the disruption of the *tqaA* gene in P. digitatum PdKH8 strain, but Δ*tqaA* mutants did not show affected virulence (35). In contrast, tryptoquialanine A inhibits citrus germlings, which attributes, to these toxins, a role in pathogenesis (34), similar to the HC toxin from the fungal maize pathogen Cochliobolus carbonum (36). Our previous data demonstrated that null *afpB* mutants did not show differences in pathogenesis (15), despite the fact that the present study suggests that this strain would produce more toxins.

A still open question is the relationship between *afpB* gene expression and protein production. In reference 15, we showed that strains harboring *afpB* under the control of the constitutive *gpdA* promoter showed a strong reduction in growth and virulence on citrus fruits. Nevertheless, AfpB production could not be determined either in these strains or the wild-type strains despite high mRNA accumulation, similar to P. chrysogenum PAFB (37). Effective AfpB production in P. digitatum was only achieved when the gene was placed under the PAF promoter from P. chrysogenum (strain PDSG420 in this study) and when the fungus was cultivated in submerged minimal medium (17). Interestingly, AfpB could never be detected for the overproducing strain grown in potato dextrose broth (PDB) under condition 5 (data not shown), despite the fact that *afpB* showed the highest induction of our study. It must be considered that *afpB* is repressed in the presence of externally added AfpB (condition 4). Taken together, our study underlines complex and not solved links between the regulation of *afpB* expression and protein production.

The AfpB protein must be internalized to exert its inhibitory activity through an energy-dependent process (20). Our results revealed a differential expression of MFS and amino acid/peptide transporters. MFSs are involved in the transport of many substances across the membrane and contribute to fungicide resistance (38). Transcriptome analysis after prochloraz treatment of susceptible and resistant strains of P. digitatum also displayed upregulation of these transporters (39). Our results definitely suggest a role of MFS in the AfpB fungus interaction. However, the high number of these transporters in the genome and their differential transcriptomic responses make it difficult to define their involvement in the fungal response to AfpB.

Variations in the expression of genes involved in cell wall integrity and degradation are natural fungal responses to antifungals. Based on this RNA-seq analysis, we previously published the downregulation of chitin synthase genes when P. digitatum is grown in the continuous presence of AfpB, and we demonstrated that myosin motor domain chitin synthases affect sensitivity to antifungal proteins (22). OuYang and collaborators also found a big influence of citral exposure in the cell wall biogenesis pathway in P. digitatum, in which repression of chitin synthase genes was shown (40). Additionally, Aspergillus giganteus AFP inhibited biosynthesis of chitin in its target fungi (41).

Regarding cell wall degradation, the endopolygalacturonase-encoding PDIG_50670 was the most repressed under conditions 4 and 5. This gene belongs to a family of carbohydrate-active enzymes which includes both endo- and exopolygalacturonases. They are responsible for pectin degradation and are involved in fungal penetration during fruit infection (42). This downregulation during the continuous treatment with AfpB or in the overproducing strain might predict a reduced infection capability, although PDSG420 shows similar pathogenicity to that of the wild type (data not shown). In this line, there are some assays in which the deletion of endopolygalacturonases caused no altered pathogenicity, and only in double mutants of endo- and exopolygalacturonases, a reduction of infection was detected, indicating a cooperative effect of these enzymes (42). At the same time, Delgado et al. identified high quantities of other endopolygalacturonase in the proteome of P. expansum treated with PgAFP (25), which might indicate different behaviors of these enzymes in response to distinct AFPs.

The TRP-encoding gene PDIG_81760 showed an interesting gene expression pattern throughout this study. Whereas the gene expression was not influenced in the absence of AfpB, it was highly induced when the fungus was continuously growing in the presence of this protein and in the overproducer. Additionally, genes upstream and downstream of PDIG_81760 were also highly induced, suggesting a gene cluster of unknown function and regulation that turned out to be activated by AfpB. Orthologs of PDIG_81760 are conserved among several fungal species (Fig. S5). However, not all of them encode an *afp*-like gene in their genomes (data not shown). Similarly, not every fungus which encodes an *afp*-like gene in its genome encodes a PDIG_81760 ortholog. For instance, the P. expansum genome that encodes three different *afp* genes (15) does not encode any PDIG_81760 homolog. Therefore, there is no correlation between the presence of AFPs and orthologs to PDIG_81760 in different fungal genomes.

Given the anionic nature of the TRP protein and its gene expression pattern in the presence of the cationic AfpB, we hypothesized that this protein was *in vivo* Kex2 processed into small TRPs and that those peptides would have a protective effect against AfpB by interaction of their opposite charges. TRP2 and TRP3 were tested *in vitro* and *in vivo* in combination with AfpB. Interestingly, none of the TRPs antagonized AfpB activity but, rather, potentiated it, particularly TRP3. This is not the first time that an anionic secreted protein from P. digitatum is shown to modulate AfpB activity. Another anionic secreted CRP known as Sca (43), which is one of the very few secreted proteins that have been functionally characterized in P. digitatum (11), was shown to potentiate AfpB activity *in vitro*, while it enhanced virulence *in vivo*. We designed fruit inoculation experiments to determine whether TRP3 would also potentiate AfpB activity *in vivo* and thus improve the previously reported weak protective activity of AfpB (4, 7, 43). However, the combination TRP3-AfpB did not improve the control of *Penicillium* decay of citrus fruits.

On the other hand, PDIG_81760 had a remarkably uncommon expression pattern during the fungal cycle. Whereas the lowest expression level was observed in liquid culture, the gene expression increased enormously at early time points during the growth in solid medium and fruit infection and suddenly declined. This “peak and decline” behavior is unique among most of the fungal genes studied during the fruit-fungus interaction, in which gene expression levels continue to increase or are maintained while infection progresses (see, e.g., Fig. 4C and references 15 and 43 to 44). This pattern would suggest an important role of PDIG_81760 in early fruit infection and disease outcome. However, null PDIG_81760 mutants did not show altered growth or sensitivity to AfpB under the conditions tested and showed only slightly delayed infection capability at early stages of fruit infection. These results do not allow us to demonstrate a role of PDIG_81760 in the pathogenicity of the fungus under the conditions tested.

ALS catalyzes the first step in the biosynthesis of acetoin and branched-chain amino acids. ALS enzymes are known targets of numerous commercial herbicides (45). ALS-inhibiting compounds have recently been proposed as novel antifungal agents (46, 47), thus emphasizing the importance of this pathway as an antifungal target. Our results additionally link the acetoin biosynthetic pathway with the activity of antifungal biomolecules such as AfpB. The genes PDIG_14840 and PDIG_14850, which encode the catalytic subunit of ALS and ALD, respectively, are highlighted for their early induction under conditions 2 and 3 and their repression under conditions 4 and 5 (Fig. 4B). Acetoin has been reported as an antifungal volatile from biocontrol agents against a variety of phytopathogens, such as B. cinerea (48, 49), and has been identified among the volatiles of effective biocontrol strains against the phytopathogens Penicillium italicum and P. digitatum (50, 51).

To test the involvement of acetoin biosynthesis in the antifungal mechanism of AfpB, we generated single and double deletion mutant strains for these two genes. Although they did not show significant differences in their virulence or growth compared to the wild type, *in vitro* antifungal activity assays demonstrated that all mutants were slightly more tolerant to AfpB (Fig. 5). These results indicate a contribution of acetoin to the antifungal effect of AfpB since preventing the fungus from producing acetoin by blocking its biosynthetic pathway diminishes the harmful effects of the protein. The abrupt change of expression of PDIG_14840 and PDIG_14850 between conditions 2 to 3 and 4 to 5 should therefore be explained by the antifungal activity of acetoin against P. digitatum and the adaptation to the presence of AfpB. AfpB treatment at early time points causes an overexpression of these genes by an unknown mechanism, leading to acetoin overproduction and subsequent deleterious effects. When cultured in the presence of sublethal concentrations of AfpB or even in the AfpB-producing strain, the fungus might adapt to the harmful effects of acetoin and repress its pathway.

In conclusion, this transcriptomic study offers a rich source of information to advance in the characterization of the biological role and multitarget mode of action of AfpB. In brief, we concluded that the *afpB* gene contributes to the overall homeostasis of the cell and that AfpB is involved in apoptotic processes and represses toxin-encoding genes in its parental fungus. Additionally, we showed that the acetoin biosynthetic pathway contributes to AfpB antifungal activity and that an extracellular TRP modulates AfpB activity. This data set will undoubtedly be the source of new hypotheses and hypothesis-testing experiments for all the community working on antifungal proteins and peptides and also for those developing new antifungals.

## MATERIALS AND METHODS

### Strains, media, and growth conditions.

The strains P. digitatum CECT 20796 (isolate PHI26) (14); P. digitatum Δ*afpB* mutant PDMG122, which is a knockout mutant in which the *afpB* gene has been partially deleted (15); and the AfpB overproducer PDSG420 (17) were used in this study. P. digitatum CECT 20796 was used as parental strain for further genetic modifications. All fungal strains and transformants were cultured on PDA plates (Difco, BD Diagnostics) for 5 to 7 days at 25°C. Conidia were harvested and dispersed in sterile Milli-Q H_2_O, and concentration was adjusted using a hemocytometer. To analyze growth on solid plates, 5 μL of conidia suspension (5 × 10^4^ conidia/mL) of parental and mutant strains were deposited in the center of PDA plates, and growth diameter was measured from 3 to 7 days. Assays were performed in technical triplicates.

Plasmids were propagated in Escherichia coli JM109 grown in Luria-Bertani (LB) medium supplemented with either 25 μg/mL chloramphenicol, 50 μg/mL kanamycin, or 100 μg/mL spectinomycin at 37°C, depending on the vector. The Agrobacterium tumefaciens AGL-1 strain used for fungal transformation was grown in LB supplemented with 20 μg/mL rifampin at 28°C.

### Improvement of P. digitatum genome annotation.

Annotation of the P. digitatum CECT 20796 genome (GenBank assembly accession no. GCA_000315665) was improved with the Functional Annotation package within the OmicsBox suite (https://www.biobam.com/omicsbox/) (52) by searching a local database with 11 fungal genomes that were phylogenetically related to P. digitatum and extensively annotated (see Table S1 in the supplemental material). We generated a local database that included all downloaded genomic assemblies except for P. digitatum CECT 20796. The NCBI BLAST+ tool was used to blast all sequences from P. digitatum against the local database with an E value similarity threshold of 1.0E-3 and default parameters. Next, GO terms associated with BLAST hits were retrieved with the mapping tool provided within OmicsBox. Mapped GO terms were selected and assigned to query sequences in an annotation step that follows the OmicsBox annotation rule. For this, the GO annotation tool was used with an annotation cutoff of 55, GO weight of 5, and an E value threshold of 1.0E-6. Multistep mapping processes, different parameters, and the annotation rule formula are further detailed in the OmicsBox manual. In order to incorporate P. digitatum UniProt IDs, gene names were mapped to their corresponding UniProt IDs in the same software. Subsequently, GO annotation was extended by performing a second annotation step with InterProScan (InterPro protein annotation) (53) included in OmicsBox. All databases of families, domains, sites, and sequence repeats were used to increase the GO terms. Finally, InterProScan GOs and previously annotated GO terms were merged to obtain the improved annotation of the P. digitatum CECT 20796 genome.

### RNA isolation and transcriptomic analysis.

For each condition (Table 1), 1× 10^6^ conidia/mL was inoculated in 1-L Erlenmeyer flasks containing 200 mL 25% PDB for 48 h at 25°C and 180 rpm. Mycelia were filtered and frozen in liquid nitrogen and stored at −80°C until RNA isolation. Total RNA was isolated using TRIzol reagent (Invitrogen, United States) following the manufacturer’s instructions. RNA concentration and quality were assessed using a spectrophotometer (NanoDrop ND-1000) and agarose gel electrophoresis.

RNA-seq analysis was performed by the genomic facility of the central service for experimental research (SCSIE) from the University of Valencia (UV, Spain) (https://www.uv.es/uvweb/central-service-for-experimental-research/en/central-service-experimental-research-scsie-1285868582594.html). Integrity of RNA samples was verified using the 2100 Bioanalyzer (Agilent Technologies, Inc., Santa Clara, USA). cDNA libraries were prepared with the TruSeq RNA sample preparation kit v2 (Illumina) and sequenced on an Illumina NextSeq 500 platform (single-end reads, 75 bp average length).

Quality (Q) of reads was checked with FastQC v0.11.3 (54). Raw data were trimmed, and low-quality reads (average Q < 20, read length < 25 bp) were filtered using Cutadapt version 1.8.3 and/or Trimmomatic 0.38 (55). Filtered reads were mapped onto the P. digitatum CECT 20796 genome (NCBI, WGS AKCT01) with TopHat2 (v2.0.10) or STAR (v2.7.8a). Further analysis was performed using software packages included in OmicsBox. The number of reads that overlapped with gene features was counted based on HTSeq 0.9.0 (56). Count data output was extracted and used for pairwise differential expression analysis with edgeR (57). Genes for which count per million (CPM) was <0.6 in a minimum of three to four samples (depending on the number of biological replicates) were removed, and library sizes were normalized using the trimmed mean of M-values (TMM) method. Differentially expressed genes (DEGs) were identified using |log_2_(fold change)| of >1 and false-discovery rate (FDR) of <0.05. The heatmap.2 function from the ggplot R package was used to generate cluster heatmaps. Multidimensional scaling plots (MDSs) were generated with the plotMDS function from the limma Bioconductor package.

GO enrichment analysis of DEGs was performed using the FatiGO package (58) based on Fisher’s exact test function (FDR < 0.05).

### Generation of P. digitatum deletion strains.

Molecular assemblies for gene disruption by homologous recombination were generated with the FungalBraid (FB) modular cloning platform (17). Plasmids and primers used are described in Tables S3 and S4, respectively.

Flanking sequences for gene disruption (around 1 kb) were cloned into the entry vector pUPD2 through restriction-ligation reactions as described (17, 59). Combinations of these sequences were assembled into the binary vector pDGB3α2 with FB parts of the hygromycin resistance marker *hph* (FB012) and the transcriptional unit for 5-fluoro-2′-deoxyuridine (F2dU) sensitivity HSV*tk* (FB013) to generate the disruption cassettes for the genes PDIG_81760 (FB091), PDIG_14840 (FB171), and PDIG_14850 (FB092) and the cassette to generate the double knockout of PDIG_14840 and PDIG_14850 (FB172). Binary vectors were introduced into A. tumefaciens AGL-1 by electroporation. P. digitatum CECT 20796 was transformed through A. tumefaciens-mediated transformation (ATMT) as described (60) with modifications (59, 61). Deletion strains were confirmed by PCR.

### Quantitative reverse transcription-PCR (qRT-PCR).

Experiments were designed with triplicate biological samples. Total RNA from (i) conidia and germ tubes, (ii) mycelium of P. digitatum CECT 20796 obtained from time course experiments in PDB or PDA, and (iii) infected fruit samples were obtained as described (62, 63). Total RNA was used to synthesize first-strand cDNA, and qRT-PCR was performed and analyzed (62). Primers used for qRT-PCR are shown in Table S4. Relative changes in gene expression were normalized using the housekeeping genes coding for P. digitatum β-tubulin, ribosomal protein L18a, and 18S rRNA simultaneously. The relative expression software tool (Multiple Condition Solver REST-MCS v2) (64) was used to determine the relative quantification of target gene expression, and statistical significance was determined using REST 2009 software (https://www.qiagen.com/es/resources/).

### Antifungal activity assays.

AfpB purification was performed as previously described (16). The tandem repeat peptides TRP2 (EEPVNELSCTNSGEMACAGIVEKR) and TRP3 (EEPVNELSCTNSGEMACAGIVE) derived from the protein encoded by PDIG_81760 were synthetically generated and purchased from GenScript (Piscataway, USA).

Antifungal activity assays were conducted in 96-well flat-bottom microtiter plates (Nunc) in a total volume of 100 μL as described (4) with some modifications. In short, 50 μL of 10% PDB containing 2× concentrated conidia (5 × 10^4^ conidia/mL) and 0.02% (wt/vol) chloramphenicol were mixed in each well with 50 μL of 2× concentrated AfpB from serial 2-fold dilution (final concentration range from 0.25 to 16 μg/mL). When AfpB was combined with the synthetic peptides, each peptide was used in a final concentration of 16 μg/mL. Samples were prepared in technical triplicates. Plates were statically incubated for 96 h at 25°C. Growth was determined every 24 h by measuring the optical density at 600 nm (OD_600_) using a plate spectrophotometer (SPECTROstar Nano; BMG Labtech); the OD_600_ mean and standard deviation (SD) were calculated. Dose-response curves were generated from measurements after 72 h. The experiments were repeated at least twice. MIC is defined as the protein concentration that completely inhibited growth in all the experiments.

### Fruit infection assays.

P. digitatum strains were inoculated on freshly harvested oranges (Citrus sinensis L. Osbeck cv. Navel, Lane Late, Navel-Barnfield, and Valencia) as described (65) with modifications. For infection assays, three replicates of five fruits were inoculated with 5 μL of conidial suspension (10^4^ conidia/mL) at four wounds around the equator. For protection assays, three replicates of five fruits were inoculated at four wounds around the equator with 5 μL of conidial suspensions (10^4^ conidia/mL), which were preincubated for 24 h with 100 μg/mL AfpB, 20 μg/mL of the synthetic TRPs, or a combination of 100 μg/mL AfpB with 20 μg/mL TRPs. In all cases, control mock inoculations were performed with 5 μL of sterile Milli-Q H_2_O. Fruits were stored at 20°C and 90% relative humidity. Each inoculated wound was scored for infection symptoms on consecutive days postinoculation (dpi). The experiments were repeated at least twice. Statistically significant differences were referred to as *P* values of <0.05 (one-way analysis of variance [ANOVA] and Tukey's honestly significant difference [HSD] test).

### Data availability.

The complete sequence of the gene PDIG_81760 was obtained by Sanger sequencing at the SCSIE (UV, Spain) using the oligonucleotides OJM614, OJM615, OJM616, and OJM617 (Table S4), and the sequence was deposited in GenBank (https://www.ncbi.nlm.nih.gov/genbank/) with accession number ON323493. Transcriptomic data generated in this study were deposited in the Gene Expression Omnibus (GEO) public database (https://www.ncbi.nlm.nih.gov/geo/) with accession numbers GSE216215 and GSE216216.
