# Supplementary material for: Transcriptomic Profile of Penicillium digitatum Reveals Novel Aspects of the Mode of Action of the Antifungal Protein AfpB
Source: Microbiol Spectr. 2023 Apr 6;11(3):e04846-22. doi: 10.1128/spectrum.04846-22 (PMC10269557; doi:10.1128/spectrum.04846-22)
Supplement: Supplemental file 1 — Supplemental material. Download spectrum.04846-22-s0001.pdf, PDF file, 1.4 MB [file spectrum.04846-22-s0001.pdf]

Supplemental Material

Transcriptomic profile of *Penicillium digitatum* reveals novel aspects of the mode of action of the antifungal protein AfpB

Carolina Ropero-Pérez[§], Begoña Bolós[§], Moisés Giner-Llorca[§], Antonella Locascio, Sandra Garrigues, Mónica Gandía[†], Paloma Manzanares and Jose F. Marcos[#]

Department of Food Biotechnology, Instituto de Agroquímica y Tecnología de Alimentos (IATA), Consejo Superior de Investigaciones Científicas (CSIC), Avda Agustín Escardino 7, 46980 Paterna, Valencia, Spain.

[§] These authors contributed equally to this work and shall be considered first co-authors. Author order has been randomly assigned.

[#] Corresponding author: Dr. Jose F. Marcos. Instituto de Agroquímica y Tecnología de Alimentos (IATA)-CSIC, Avenida Agustín Escardino 7, Paterna 46980, Valencia, Spain. e-mail: jmarcos@iata.csic.es. Phone: (34)963.900.022. Fax: (34)963.636.301

[†] Present address: Departamento de Medicina Preventiva y Salud Pública, Ciencias de la Alimentación, Toxicología y Medicina Legal. Facultad de Farmacia. Universitat de València. Vicente Andrés Estellés s/n, Burjassot 46100, Valencia, Spain.

Supplemental Table S1. List of organisms and their genome assembly obtained from NCBI used to improve the functional annotation of *P. digitatum* CECT 20796 (PHI26).

Species	Assembly	GenBank/RefSeq assembly accession	PMID	Ref
Penicillium chrysogenum	ASM71027v1	GCA_000710275.1	25059858	(1)
Penicillium expansum	ASM76974v1	GCF_000769745.1	25338147	(2)
Aspergillus fumigatus	ASM265v1	GCF_000002655.1	16372009	(3)
Aspergillus nidulans	ASM14920v2	GCF_000149205.2	16372000	(4)
Aspergillus niger	ASM285v2	GCF_000002855.3	18318841	(5)
Botrytis cinerea	ASM14353v4	GCF_000143535.2	26913498	(6)
Fusarium oxysporum	FO_FOSC_3_a_V1	GCF_000271745.1	-	-
Magnaporthe oryzae	MG8	GCF_000002495.2	15846337	(7)
Neurospora crassa	NC12	GCF_000182925.2	12712197	(8)
Penicillium digitatum	ASM1676781v1	GCA_016767815.1	-	-
Penicillium italicum	ASM76976v1	GCA_000769765.1	25338147	(2)

References:

1. Specht T, Dahlmann TA, Zadra I, Kürnsteiner H, Kück U. 2014. Complete sequencing and chromosome-scale genome assembly of the industrial progenitor strain P2niaD18 from the penicillin producer *Penicillium chrysogenum*. Genome Announc 2.
2. Ballester AR, Marcet-Houben M, Levin E, Sela N, Selma-Lázaro C, Carmona L, Wisniewski M, Droby S, González-Candelas L, Gabaldón T. 2015. Genome, transcriptome, and functional analyses of *Penicillium expansum* provide new insights into secondary metabolism and pathogenicity. Mol Plant Microbe Interact 28:232-48.
3. Nierman WC, Pain A, Anderson MJ, Wortman JR, Kim HS, Arroyo J, Berriman M, Abe K, Archer DB, Bermejo C, Bennett J, Bowyer P, Chen D, Collins M, Coulsen R, Davies R, Dyer PS, Farman M, Fedorova N, Fedorova N, Feldblyum TV, Fischer R, Fosker N, Fraser A, García JL, García MJ, Goble A, Goldman GH, Gomi K, Griffith-Jones S, Gwilliam R, Haas B, Haas H, Harris D, Horiuchi H, Huang J, Humphray S, Jiménez J, Keller N, Khouri H, Kitamoto K, Kobayashi T, Konzack S, Kulkarni R, Kumagai T, Lafton A, Latgé J-P, Li W, Lord A, Lu C, et al. 2005. Genomic sequence of the pathogenic and allergenic filamentous fungus *Aspergillus fumigatus*. Nature 438:1151-1156.
4. Galagan JE, Calvo SE, Cuomo C, Ma LJ, Wortman JR, Batzoglou S, Lee SI, Baştürkmen M, Spevak CC, Clutterbuck J, Kapitonov V, Jurka J, Scazzocchio C, Farman M, Butler J, Purcell S, Harris S, Braus GH, Draht O, Busch S, D'Enfert C, Bouchier C, Goldman GH, Bell-Pedersen D, Griffiths-Jones S, Doonan JH, Yu J, Vienken K, Pain A, Freitag M, Selker EU, Archer DB, Peñalva MA, Oakley BR, Momany M, Tanaka T, Kumagai T, Asai K, Machida M, Nierman WC, Denning DW, Caddick M, Hynes M, Paoletti M, Fischer R, Miller B, Dyer P, Sachs MS, Osmani SA, Birren BW. 2005. Sequencing of *Aspergillus nidulans* and comparative analysis with *A. fumigatus* and *A. oryzae*. Nature 438:1105-15.
5. Pel HJ, de Winde JH, Archer DB, Dyer PS, Hofmann G, Schaap PJ, Turner G, de Vries RP, Albang R, Albermann K, Andersen MR, Bendtsen JD, Benen JAE, van den Berg M, Breestraat

S, Caddick MX, Contreras R, Cornell M, Coutinho PM, Danchin EGJ, Debets AJM, Dekker P, van Dijck PWM, van Dijk A, Dijkhuizen L, Driessen AJM, d'Enfert C, Geysens S, Goosen C, Groot GSP, de Groot PWJ, Guillemette T, Henrissat B, Herweijer M, van den Hombergh JPTW, van den Hondel CAMJJ, van der Heijden RTJM, van der Kaaij RM, Klis FM, Kools HJ, Kubicek CP, van Kuyk PA, Lauber J, Lu X, van der Maarel MJEC, Meulenberg R, Menke H, Mortimer MA, Nielsen J, Oliver SG, et al. 2007. Genome sequencing and analysis of the versatile cell factory *Aspergillus niger* CBS 513.88. Nature Biotechnol 25:221-231.

6. Van Kan JA, Stassen JH, Mosbach A, Van Der Lee TA, Faino L, Farmer AD, Papasotiriou DG, Zhou S, Seidl MF, Cottam E, Edel D, Hahn M, Schwartz DC, Dietrich RA, Widdison S, Scalliet G. 2017. A gapless genome sequence of the fungus *Botrytis cinerea*. Mol Plant Pathol 18:75-89.
7. Dean RA, Talbot NJ, Ebbole DJ, Farman ML, Mitchell TK, Orbach MJ, Thon M, Kulkarni R, Xu J-R, Pan H, Read ND, Lee Y-H, Carbone I, Brown D, Oh YY, Donofrio N, Jeong JS, Soanes DM, Djonovic S, Kolomiets E, Rehmeyer C, Li W, Harding M, Kim S, Lebrun M-H, Bohnert H, Coughlan S, Butler J, Calvo S, Ma L-J, Nicol R, Purcell S, Nusbaum C, Galagan JE, Birren BW. 2005. The genome sequence of the rice blast fungus *Magnaporthe grisea*. Nature 434:980-986.
8. Galagan JE, Calvo SE, Borkovich KA, Selker EU, Read ND, Jaffe D, FitzHugh W, Ma L-J, Smirnov S, Purcell S, Rehman B, Elkins T, Engels R, Wang S, Nielsen CB, Butler J, Endrizzi M, Qui D, Ianakiev P, Bell-Pedersen D, Nelson MA, Werner-Washburne M, Selitrennikoff CP, Kinsey JA, Braun EL, Zelter A, Schulte U, Kothe GO, Jedd G, Mewes W, Staben C, Marcotte E, Greenberg D, Roy A, Foley K, Naylor J, Stange-Thomann N, Barrett R, Gnerre S, Kamal M, Kamvysselis M, Mauceli E, Bielke C, Rudd S, Frishman D, Krystofova S, Rasmussen C, Metzenberg RL, Perkins DD, Kroken S, et al. 2003. The genome sequence of the filamentous fungus *Neurospora crassa*. Nature 422:859-868.

Supplemental Table S2. RNA-Seq stats for Experiment I and II sample reads.

		Processed reads	Mapped reads	% mapped reads	Reads mapped to transcripts	% mapped reads to transcripts
**Experiment I**	A1	29787538	26047801	87.45	19724825	75.73
	A2	25236596	22242410	88.14	16593847	74.60
	A3	26478870	22823236	86.2	17091531	74.89
	A4	27120849	24097558	88.85	18416676	76.43
	B1	28076281	23652476	84.24	17910005	75.72
	B2	26853161	23480270	87.44	17571617	74.84
	B3	35095222	31115749	88.66	23827223	76.58
	B4	29341169	26799911	91.34	20406194	76.14
**Experiment II**	1A	33258488	31016180	93.3	20568280	66.31
	1B	28980317	27147855	93.7	19610962	72.24
	1C	22740015	20935103	92.1	14900386	71.17
	2A	24555108	22915209	93.3	16619102	72.52
	2B	40613625	37288336	91.8	25716069	68.97
	2C	36282399	34070114	93.9	23941690	70.27
	3A	33138400	30729838	92.7	21970201	71.49
	3B	28944386	26744483	92.4	19316593	72.23
	3C	33298233	31177915	93.6	21894138	70.22
	4A	34958519	32854821	94	26070138	79.35
	4B	27054052	25329369	93.6	20128189	79.47
	4C	25110404	23478951	93.5	18378926	78.28
	5A	31941085	29944200	93.7	22441156	74.94
	5B	21798004	23915586	92.4	15620764	65.32
	5C	25739493	23915586	92.9	18270262	76.39

Supplemental Table S3. FungalBraid (FB) elements used and FB vectors generated in this study.

Code	Genetic Element(s)	FB Plasmid	Reference
FB012	*PtrpC::hph::Ttub*	pUPD2	(1)
FB013	*PgpdA::HSVtk::Ttub*	pUPD2	(1)
FB087	PDIG_14850 5' flanking sequence	pUPD2	This work
FB088	PDIG_14850 3' flanking sequence	pUPD2	This work
FB089	PDIG_81760 5' flanking sequence	pUPD2	This work
FB090	PDIG_81760 3' flanking sequence	pUPD2	This work
FB091	FB013::FB089::FB012::FB090	pDGBα2	This work
FB092	FB013::FB087::FB012::FB088	pDGBα2	This work
FB168	PDIG_14840 5' flanking sequence	pUPD2	This work
FB169	PDIG_14840 3' flanking sequence	pUPD2	This work
FB171	FB013::FB168::FB012::FB169	pDGBα2	This work
FB172	FB013::FB168::FB012::FB088	pDGBα2	This work

References:

1. Hernanz-Koers M, Gandía M, Garrigues S, Manzanares P, Yenush L, Orzaez D, Marcos JF. 2018. FungalBraid: A GoldenBraid-based modular cloning platform for the assembly and exchange of DNA elements tailored to fungal synthetic biology. Fungal Genet Biol 116:51-61.

Supplemental Table S4. Primers used in this study.

Name	F/R	Sequence 5´→3´	Origin	Use	Reference
OJM85	F	AGCGGTGACAAGTACGTTCC	*β-tub*	qPCR	(1)
OJM86	R	ACCCTTGGCCCAGTTGTTAC	*β-tub*	qPCR	(1)
OJM151	F	TGGGGCAGAGGGAACTTGAG	*L18a*	qPCR	(2)
OJM152	R	ACCGACGCTGTTGAGGCTCT	*L18a*	qPCR	(2)
OJM232	R	GTTTGCCAGTGATACACATGGG	*hph*	PCR	(3)
OJM334	F	CGACTTCAGGAAGGGGTGTA	*18S rRNA*	qPCR	(4)
OJM335	R	CTTGGATGTGGTAGCCGTT	*18S rRNA*	qPCR	(4)
OJM477	R	CGCCATGTAGTGTATTGACCG	*hph*	PCR	This work
OJM604	F	CATATCCAAGCCATTCGTCTCC	*PDIG_81760*	PCR	This work
OJM605	R	GGTTCTTACACTTCTGAATTCC	*PDIG_81760*	PCR	This work
OJM611	R	CGTTTGGCGAGGTCTACTCCC	*PDIG_14850*	PCR	This work
OJM612	F	CAGTATTCGGTTCTTAGCGC	*PDIG_14850*	qPCR/PCR	This work
OJM613	R	GCCTGGAGGAATCTGGTAGG	*PDIG_14850*	qPCR	This work
OJM614	F	ATGAAGTCTCAAACCGTCCTC	*PDIG_81760*	qPCR/Seq	This work
OJM615	R	TCAATTGGAGCGGCCATGAC	*PDIG_81760*	qPCR/PCR/Seq	This work
OJM616	F	CGATCCTCCATCTCTTTCCCGC	*PDIG_81760*	Seq	This work
OJM617	R	GCTGGATCTATACAAAACTCC	*PDIG_81760*	Seq	This work
OJM626	F	TACTTGCCTGTGCTGATCCT	*PDIG_14840*	PCR	This work
OJM627	R	CTTCATCATTACAGCACCGCG	*PDIG_14840*	PCR	This work
OJM648	F	GACGGCACGCCAATGATTGTG	*PDIG_14840*	qPCR	This work
OJM649	R	GTCGCCAATTCTCTCCACAGC	*PDIG_14840*	qPCR	This work

References:

1. Sanzani SM, Schena L, Nigro F, De Girolamo A, Ippolito A. 2009. Effect of quercetin and umbelliferone on the transcript level of *Penicillium expansum* genes involved in patulin biosynthesis. Eur J Plant Pathol 125:223-233.
2. Gandía M, Harries E, Marcos JF. 2012. Identification and characterization of chitin synthase genes in the postharvest citrus fruit pathogen *Penicillium digitatum*. Fungal Biol 116:654-664.
3. Gandía M, Harries E, Marcos JF. 2014. The myosin motor domain-containing chitin synthase PdChsVII is required for development, cell wall integrity and virulence in the citrus postharvest pathogen *Penicillium digitatum*. Fungal Genet Biol 67:58-70.
4. Nair R, Roy I, Bucke C, Keshavarz T. 2009. Quantitative PCR study on the mode of action of oligosaccharide elicitors on penicillin G production by *Penicillium chrysogenum*. J Appl Microbiol 107:1131-9.

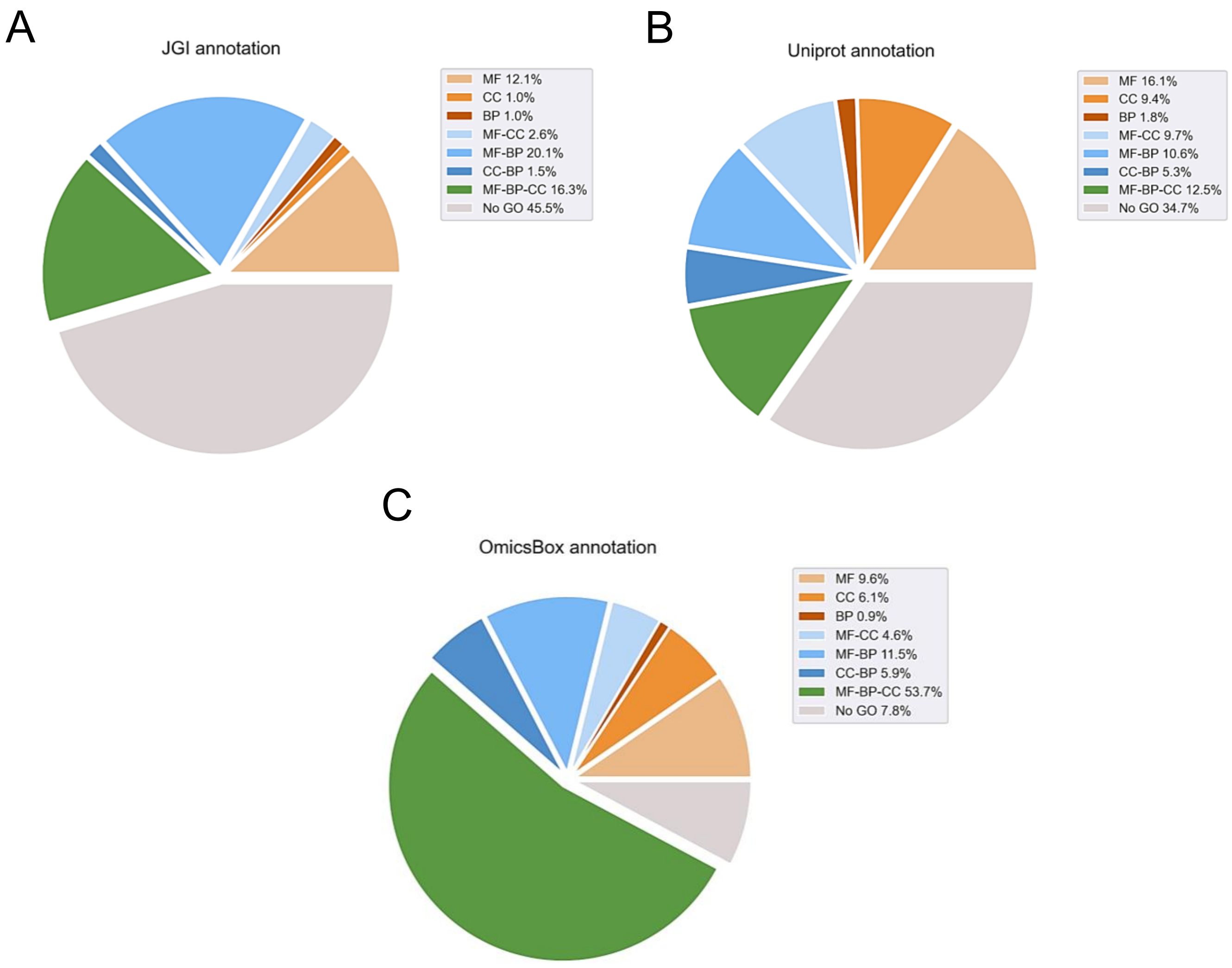

Supplemental Fig. S1: Comparison of *P. digitatum* CECT 20796 functional annotation between different public databases and the OmicsBox annotation. GO term categories referring to molecular function (MF), cellular component (CC) and biological process (BP) were used to analyze differences between the functional annotation present in public databases such as JGI database (A), Uniprot database (B) and OmicsBox annotation result (C). Proteins were grouped into one single GO term category or different categories which present a combination of two, three or none of the above mentioned GO term categories.

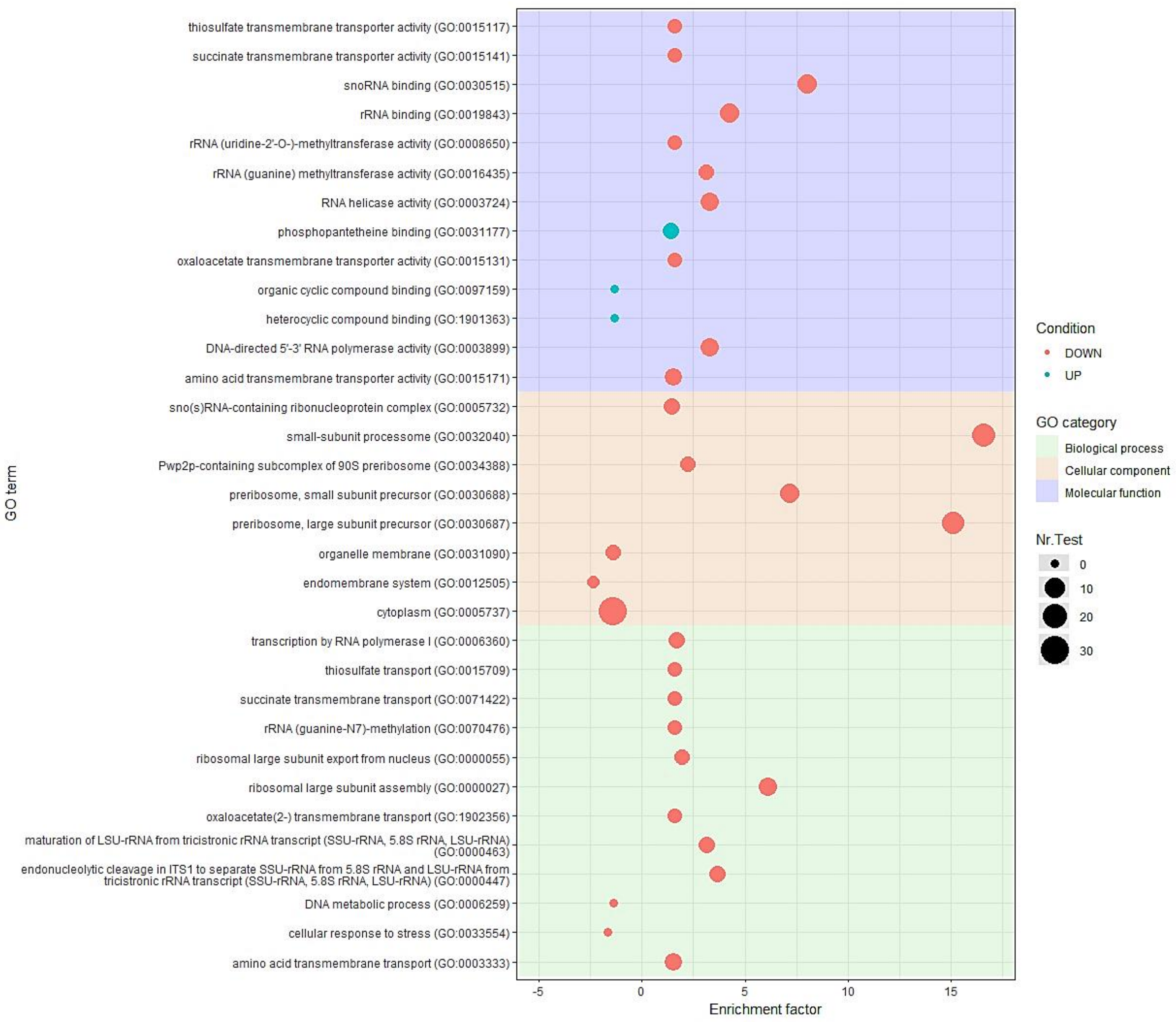

Supplemental Fig. S2. Gene Ontology (GO) enrichment analysis for the differentially expressed genes (DEGs) in the Δ*afpB* mutant (PDMG122). The y-axis indicates the GO term and the x-axis shows the enrichment factor calculated as $-\log_{10}$(FDR) for over-represented categories and $\log_{10}$(FDR) for under-represented categories (FDR<0.05). The bubble size represents the number of DEGs detected for each GO term. The color background differentiates the GO category and the bubble colors distinguish up-regulated (blue) and down-regulated (red) genes.

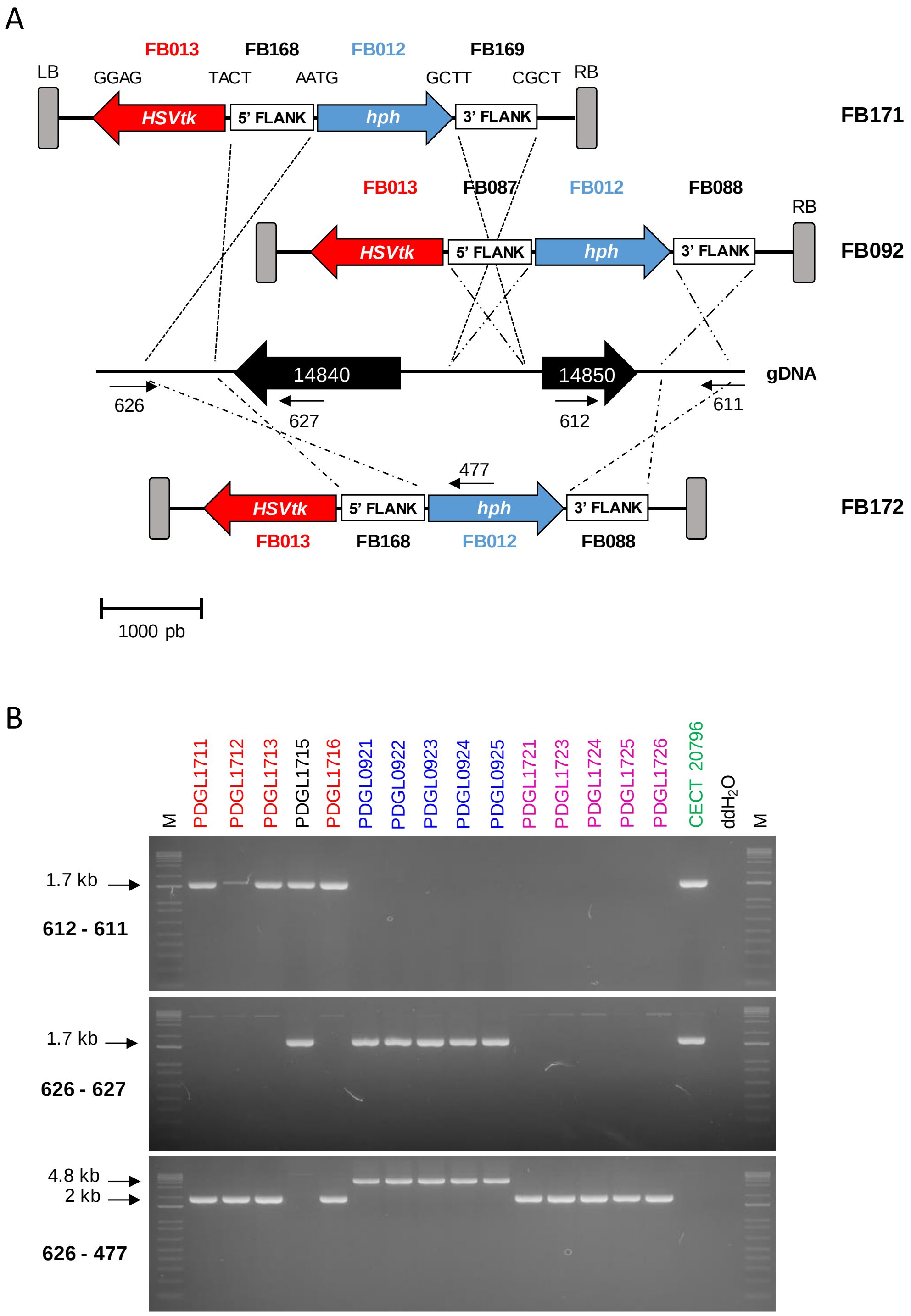

Supplemental Fig. S3. Generation of *P. digitatum* PDIG_14840 and PDIG_14850 single and double deletion strains and confirmation by PCR. (A) Schematic representation of PDIG_14840 and PDIG_14850 genes in the parental strain CECT 20796 and the FB constructs used for single (FB092 and FB171) and double (FB172) deletion. All primers used for PCR analysis are localized in the figure. (B) PCR amplification of genomic DNA of the distinct *P. digitatum* strains with different primer pairs as indicated. The PDIG_14850 deletion mutants (PDGL0921 to PDGL0925) and the double deletion mutants (PDGL1721 to PDGL1726) did not show any amplicon with primers 612/611, while PDIG_14840 deletion strains (PDGL1711 to PDGL1714, and PDGL1716), the ectopic transformant PDGL1715 and the parental strain showed an amplicon of 1.7 kb (first panel). The PDIG_14840 deletion mutants and the double deletion mutants showed no amplicon with primers 626/627, while the PDIG_14850 deletion mutants, the ectopic transformant and the parental strain showed an amplicon of 1.7 kb (second panel). All the positive transformants showed an amplicon with primers 626/477 indicating the presence of the hygromycin marker (third panel).

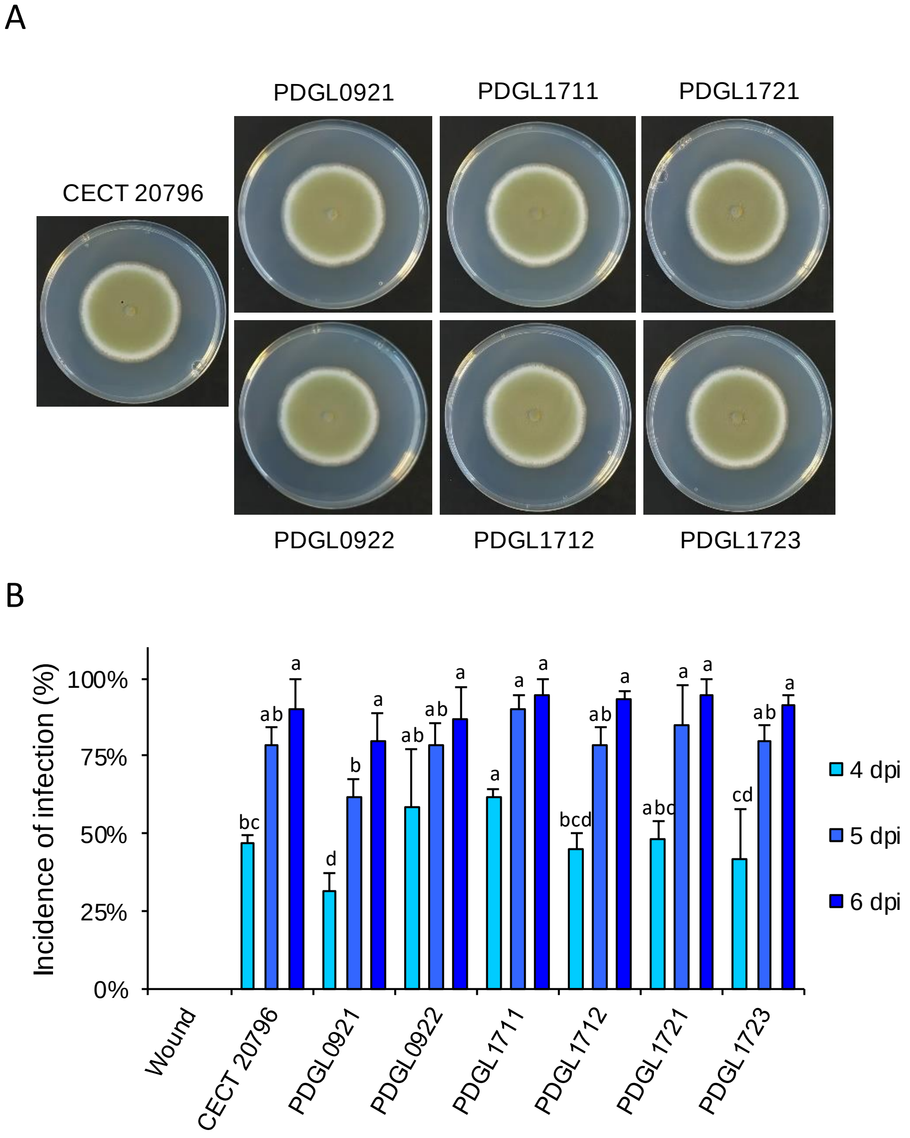

Supplemental Fig. S4. **Phenotypical characterization of *P. digitatum* deletion strains for the acetoin biosynthesis pathway-related genes**. (A) Representative images of growth on PDA plates of *P. digitatum* CECT 20796 (PHI26), the deletion strains for PDIG_14850 (PDGL0921 and PDGL0922) PDIG_14840 (PDGL1711 and PDGL1712) and the double deletion strains (PDGL1721 and PDGL1723). (B) Infection of orange fruits cv Navel by *P. digitatum* CECT 20796 and the deletion strains. Bars show the mean values of the percentage of infected wounds and standard deviation (SD) of three replicates of five oranges at 4, 5 and 6 dpi. Letters show significant differences among the treatments at each independent day (one-way ANOVA and Tukey's HSD test, $p < 0.05$).

```
Pdig PHI26 (ON323493)          -------------MKSQTVLSIL-----FAACSVMAAPIDTPVNELSCTNSGEMACAGIV 42
Pdig PDW03 (KAG0155198.1)      -------------MKSQTVLSIL-----FAACSVMAAPIDTPVNELSCTNSGEMACAGIV 42
Pgri (XP_040653216.1)          -------------MKSQTILSIL-----FAACSVMAAPIDTQVNELACTKSGDMACSGVV 42
Pvul (OQE05931.1)              -------------MKSQAVLSVL-----FAACSVMAAPIDNQINELACTLSGDMACAGVV 42
Csal (KXH34237.1)              -------------MYFSVALVAGIVASATAIPANSARTVDTVANELACVARGDMACAGLV 47
Casi (KAF0325118.1)            MVDLQIRHPNSVIVRYSIAFVAGIAASVTAIPTEPARNVDTVSNELACVARGDMACAGLV 60
Cglo (KAH9238470.1)            -------------MRYSIAFVAGIAASITAIPTEPARNVDTVANELACVARGDMACAGLV 47
Cfio (XP_053048972.1)          -------------MHISMALVAGIAACAAAIPVNSARTIDTVANELACVARGDMACAGIA 47
Tlen (KAF3073163.1)            -------------MRFSTILIAATGAVA---ASLPARTVENVANELACVARGDMACAGLV 44
                                            :  .  :               *  ::.  ***:*.  *:***:*:.

Pdig PHI26 (ON323493)          EKREEP--VNELSCTNSGEMACAGIVEKREE---P-VNELSCTNSGEMACAGIVEKRE-- 94
Pdig PDW03 (KAG0155198.1)      EKREEP--VNELSCTNSGEMACAGIVEKREE---P-VNELSCTNSGEMACAGIVEKRE-- 94
Pgri (XP_040653216.1)          EKREEL--VNELACTKSGDMACSGVVEKREE---L-VNELACTKSGDMACSGVVEKRE-- 94
Pvul (OQE05931.1)              EKREEPE-INELACTLSGDMACAGVVEKREE---PEINELACTLSGDMACAGVVEKRE-- 96
Csal (KXH34237.1)              SKRSEAELANELACVARGDMACAGLVEKRDPEMEQLANELSCVARGDMACAGLVSKKRAV 107
Casi (KAF0325118.1)            SKRSEDELANELACVARGDMACAGLVAKRDPEMEQLANELACVARGDMACAGLVSKKRAV 120
Cglo (KAH9238470.1)            SKRSENELANELACVARGDMACAGLVAKRDPEMEQLADELACVARGDMACAGLVSKKRAV 107
Cfio (XP_053048972.1)          SKRTATDLANELACVARGDMACAGHVEKRDPEIEQLANELSCVARGDMACAGLASQKRAV 107
Tlen (KAF3073163.1)            GKRSADEIANELACVARGDMACSGLVSKRGS--DSVANELACVARGDMACAGLVSKKRAT 102
                                **      ***:*.  *:***:* * **        :**:*.  *:***:*:..::.

Pdig PHI26 (ON323493)          E-PVNELSCTNSGEMACAGIVEKREE-P-VNELSCTNSGEMACAGIVEKRE---EPVNEL 148
Pdig PDW03 (KAG0155198.1)      E-PVNELSCTNSGEMACAGIVEKREE-P-SMS-CLHNSGEMACAGIVEKRE---EPVNEL 147
Pgri (XP_040653216.1)          E-LVNELACTKSGDMACSGVVEKREE-L-VNELACTKSGDMACSGVVEKRE---ELVNEL 148
Pvul (OQE05931.1)              ESEINELACTLSGDMACAGVVEKREE-PEINELACTLSGDMACAGVVEKRE--------- 146
Csal (KXH34237.1)              EAAINELSCVARGDMACAGLVAKRN-ADSINELSCVARGDMACAGLVAKKRDTADVANEL 166
Casi (KAF0325118.1)            ERAINELACTASGDMACAGPVSKRASPEDVNELACVARGDMACAGLVAKKRDTTDVANEL 180
Cglo (KAH9238470.1)            ERAINELACTASGDMACAGPVSKRASPEDVNELACVARGDMACAGLVAKKRDTTDVANEL 167
Cfio (XP_053048972.1)          EAAINKLASDAQPDGACLL-NAKSISTDFSNELCCAAQGDMACAGLVAKKRDTAEVANEL 166
Tlen (KAF3073163.1)            ENAVNELACVARGDMACAGLVGKRS-PEEINELACAARGDMACAGAVAKKRSTAEVANEL 161
                               *  :*:*:.    : **     *        . .    *:***:* * *:.

Pdig PHI26 (ON323493)          SCTNSGEMACAGIVEKREEPVNELSCTNSGEMACAGIVEKREEPVNELSCTNSGEMACAG 208
Pdig PDW03 (KAG0155198.1)      SCTNSGEMACAGIVEKREEPVNELSCTNSGEMACAGIVEKREEPVNELSCTNSGEMACAG 207
Pgri (XP_040653216.1)          ACTKSGDMACSGVVE--------------------------------------------- 163
Pvul (OQE05931.1)              ------------------------------------------------------------ 146
Csal (KXH34237.1)              SCVARGDMACAGLVA--------------------------------------------- 181
Casi (KAF0325118.1)            SCVARGDMACAGVSFDVPTT---------------------------------------- 200
Cglo (KAH9238470.1)            SCVARGDMACAGLVA--------------------------------------------- 182
Cfio (XP_053048972.1)          ACVARGDMACAGVVA--------------------------------------------- 181
Tlen (KAF3073163.1)            ACVARGDMACSGLVA--------------------------------------------- 176


Pdig PHI26 (ON323493)          IVEKREEPINELSCTNSGEMACAGIVE    235
Pdig PDW03 (KAG0155198.1)      IVEKREEPINELSCTNSGEMACAGIVE    234
Pgri (XP_040653216.1)          ---------------------------    163
Pvul (OQE05931.1)              ---------------------------    146
Csal (KXH34237.1)              ---------------------------    181
Casi (KAF0325118.1)            ---------------------------    200
Cglo (KAH9238470.1)            ---------------------------    182
Cfio (XP_053048972.1)          ---------------------------    181
Tlen (KAF3073163.1)            ---------------------------    176
```

Supplemental Fig. S5. **Protein alignment of translated proteins similar to PDIG_81760**. BLASTp Protein Similarity Search to PDIG_81760 was conducted with the NCBI online tool against the non-redundant (nr) protein sequences database (https://blast.ncbi.nlm.nih.gov/Blast.cgi), and similar proteins were aligned with Clustal Omega using MEGA v 7.0.26. Fungi and the corresponding NCBI sequence ID are as follows: Pdig PHI26 (ON323493, the corrected PDIG_81760 from *Penicillium digitatum* strain PHI26 characterized in this work), Pdig PDW03 (KAG0155198.1 from *Penicillium digitatum* strain PDW03), Pgri (XP_040653216.1 from *Penicillium griseofulvum*); Pvul (OQE05931.1 from *Penicillium vulpinum*), Csal (KXH34237.1 from *Colletotrichum salicis*), Casi (KAF0325118.1 from *Colletotrichum asianum*), Cglo (KAH9238470.1 from *Colletotrichum gloeosporioides*), Cfio (XP_053048972.1 from *Colletotrichum fioriniae*), Tlen (KAF3073163.1 from *Trichoderma lentiforme*). The conserved repetitions of 20-amino acid sequence are highlighted in yellow. Different amino acids in the sequence from *Penicillium digitatum strain* PDW03 are highlighted in purple.

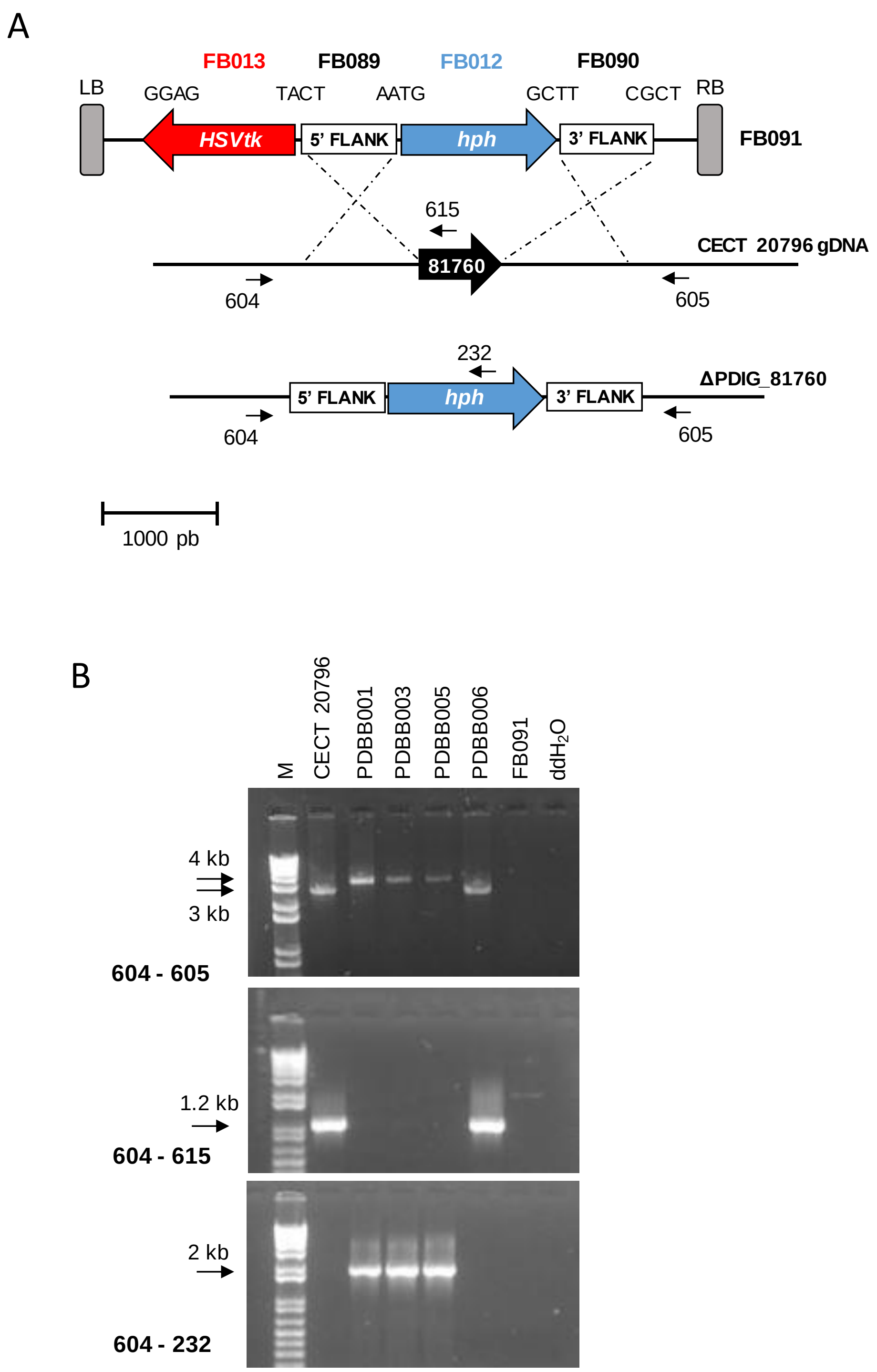

Supplemental Fig. S6. Generation of *P. digitatum* PDIG_81760 single deletion strains and confirmation by PCR. (A) Schematic representation of PDIG_81760 in the parental strain CECT 20796 and the FB construct (FB091) used for single deletion. All primers used for PCR analysis are localized in the figure. (B) PCR amplification of genomic DNA of the distinct *P. digitatum* strains with different primer pairs as indicated. The PDIG_81760 deletion mutants (PDBB001, PDBB003 and PDBB005) showed different amplicon size compared with CECT 20796 or PDBB006 ectopic strain with external primers 604/605, indicating hygromycin cassette replacement (first panel). The PDIG_81760 deletion mutants showed no amplicon with primers 604/615, where 615 was an internal primer from PDIG_81760 gene. Only CECT 20796 parental strain and PDBB006 ectopic strain showed an amplicon of expected size with these primer pair (second panel). All the positive transformants showed an amplicon with primers 604/232 indicating the presence of the hygromycin resistance cassette (third panel). Note that PDIG_81760 is in reverse orientation in the CECT 20796 genome.

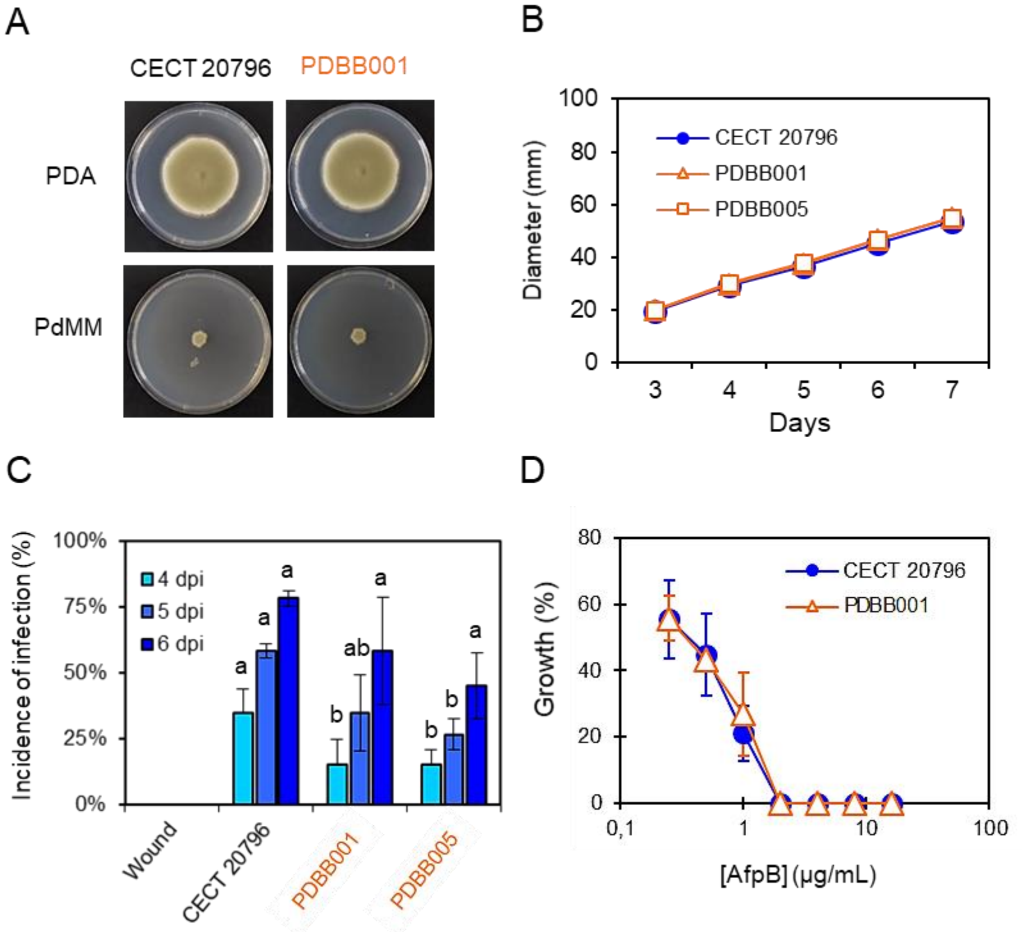

Supplemental Fig. S7. **Phenotypical characterization of *P. digitatum* PDIG_81760 deletion strains (PDBB001 and PDBB005) and infection assays on citrus fruits**. (A) Representative images of growth on PDA and PdMM plates of *P. digitatum* parental strain CECT 20796 and PDBB001. (B) Colony diameter of PDBB001 and PDBB005 transformant strains from 3 to 7 days of growth at 25 ºC in solid PDA medium, compared to parental CECT 20796 strain. (C) Infection of orange fruits cv Lane Late by three different strains. Bars show the mean values of the percentage of infected wounds and standard deviation (SD) of three replicates of five oranges at 4, 5 and 6 days post infection (dpi). Letters show significant differences among the different strains at each independent day (one-way ANOVA and Tukey's HSD test, $p < 0.05$). (D) Dose-response curves showing the antifungal activity of AfpB against *P. digitatum* wild-type strain CECT 20796 and the deletion mutant strain PDBB001.
